# Apolipoprotein-E deficiency leads to brain network alteration characterized by diffusion MRI and graph theory

**DOI:** 10.3389/fnins.2023.1183312

**Published:** 2023-11-21

**Authors:** Margaret Caroline Stapleton, Stefan Paul Koch, Devin Raine Everaldo Cortes, Samuel Wyman, Kristina E. Schwab, Susanne Mueller, Christopher Gordon McKennan, Philipp Boehm-Sturm, Yijen Lin Wu

**Affiliations:** ^1^Department of Developmental Biology, School of Medicine, University of Pittsburgh, Pittsburgh, PA, United States; ^2^Rangos Research Center Animal Imaging Core, Children’s Hospital of Pittsburgh of UPMC, Pittsburgh, PA, United States; ^3^Charité 3R | Replace, Reduce, Refine, Charité-Universitätsmedizin Berlin, corporate member of Freie Universität Berlin and Humboldt-Universität zu Berlin, Berlin, Germany; ^4^Department of Experimental Neurology and Center for Stroke Research Berlin, Charité-Universitätsmedizin Berlin, Berlin, Germany; ^5^NeuroCure Cluster of Excellence and Charité Core Facility 7T Experimental MRIs, Charité-Universitätsmedizin Berlin, Berlin, Germany; ^6^Department of Bioengineering, Swanson School of Engineering, University of Pittsburgh, Pittsburgh, PA, United States; ^7^Department of Statistics, University of Pittsburgh, Pittsburgh, PA, United States

**Keywords:** apolipoprotein-E, diffusion tractography, DTI, MRI, connectome

## Abstract

Late-onset Alzheimer’s disease (LOAD) is a major health concern for senior citizens, characterized by memory loss, confusion, and impaired cognitive abilities. Apolipoprotein-E (ApoE) is a well-known risk factor for LOAD, though exactly how ApoE affects LOAD risks is unknown. We hypothesize that ApoE attenuation of LOAD resiliency or vulnerability has a neurodevelopmental origin *via* changing brain network architecture. We investigated the brain network structure in adult ApoE knock out (ApoE KO) and wild-type (WT) mice with diffusion tensor imaging (DTI) followed by graph theory to delineate brain network topology. Left and right hemisphere connectivity revealed significant differences in number of connections between the hippocampus, amygdala, caudate putamen and other brain regions. Network topology based on the graph theory of ApoE KO demonstrated decreased functional integration, network efficiency, and network segregation between the hippocampus and amygdala and the rest of the brain, compared to those in WT counterparts. Our data show that brain network developed differently in ApoE KO and WT mice at 5 months of age, especially in the network reflected in the hippocampus, amygdala, and caudate putamen. This indicates that ApoE is involved in brain network development which might modulate LOAD risks *via* changing brain network structures.

## 1 Introduction

Apolipoprotein E (ApoE), a class of lipoproteins responsible for cholesterol transport and lipoprotein metabolism (Mahley et al., 2009), is the strongest genetic risk factor for the late-onset Alzheimer Disease (LOAD) (Biffi et al., 2010; Yu et al., 2014; Yamazaki et al., 2016; Hersi et al., 2017; Kanatsu and Tomita, 2017; Liao et al., 2017) and emerging therapeutic targets for it (Safieh et al., 2019; Uddin et al., 2019; Yamazaki et al., 2019). The 3 polymorphic alleles of ApoE, ε2, ε3, and ε4, exhibit differential effects on LOAD risks in an isoform-specific manner (Munoz et al., 2019): ApoE3 is the most common allele in the population; ApoE4 variant is found in more than 65% of AD (Heffernan et al., 2016) with 12 folds increase in occurrence of AD (Kim et al., 2014; Liao et al., 2017); whereas ApoE2 is under-represented in AD patients and thought to be neuroprotective (Dorey et al., 2017). However, how ApoE modulates risks for LOAD is not well understood.

Our study explores the possibility of neurodevelopmental origin of ApoE-imposed vulnerability or resiliency through brain network remodeling. Network dysfunctions (Palop and Mucke, 2016; Kazim et al., 2021; Pini et al., 2022) are known to be associated with LOAD and frontotemporal dementia (Filippi et al., 2013). The “dual-hit hypothesis” (Zhu et al., 2004, 2007) is well recognized in LOAD. The manifestation of the disease is synergistically caused by the “first hit,” the intrinsic vulnerability, followed by the “second hit” of a trigger later in life. We hypothesize that ApoE attenuation of LOAD resiliency or vulnerability has a neurodevelopmental origin *via* changing brain network architecture. We hypothesize that ApoE is involved in brain network development, such that when it is knocked out, it could alter the network architecture, in what could be considered a “first-hit.” This disruption from the default brain network could attenuate vulnerability or resiliency for the “second-hit” later in life, therefore affecting LOAD risks. This notion is supported by the fact that ApoE is found to be associated with synaptic plasticity (Yu et al., 2014). ApoE functions in the transportation and clearance of cholesterol in the brain, which is necessary to form and develop synapses and dendrites (Mauch et al., 2001; Hauser et al., 2011). ApoE-containing lipoproteins are able to deliver cholesterol to neurons then increasing synapse formations by aiding in the creation of synaptic vesicles (Hauser et al., 2011). Consequently, disruption in this transport of cholesterol *via* ApoE can compromise synaptic formation, impacting synaptic plasticity or resulting in synaptic and dendrite degeneration (Hauser et al., 2011). The global alteration of synaptic plasticity during neurodevelopment might contribute to changes in brain network development.

We tested this hypothesis using diffusion MRI followed by graph theory to characterize brain network topology in ApoE knock out (ApoE KO) mice compared to that of their wild-type (WT) counterparts. To test the neurodevelopmental origin of brain network alteration due to ApoE deficiency, the age of 5 months, equivalent to human younger mature adults (Hagan, 2017; Sukoff Rizzo and Crawley, 2017), was chosen when the adult mouse brain network has formed but not yet near the age of LOAD onset in aging mice (~ 18 months of age). The objective was to determine whether knocking out ApoE could result in altered brain network structure in adult mice, long before the equivalent age of LOAD onsets.

Mouse models of human disease are valuable for studying disease etiology, and imaging modalities are useful for phenotyping mice (Wu and Lo, 2017). The use of ApoE KO mice was first published in 1992 and has since been a useful tool in studying the effects of ApoE on neurodevelopment (Getz and Reardon, 2016). A number of studies showed behavioral differences in ApoE KO animals, including deficient olfactory function (Nathan et al., 2004), impaired working memory process (Gordon et al., 1995), and decreased exploratory behavior (Fuentes et al., 2018). These behavioral deficits are consistent with some LOAD symptoms, such as memory loss, impaired decision-making ability and increased emotional reactivity (How Alzheimer's disease changes the brain, 2022).

Our approach to delineate brain network structure uses diffusion tensor imaging (DTI) with subsequent graph theoretical analysis for network topology characterization. DTI is a three-dimensional MRI imaging modality that leverages local water diffusion properties to probe neuronal fiber structures based on the signal intensity following sequential gradient pulses in that voxel (Basser and Pierpaoli, 1996; Pierpaoli et al., 1996; Medina and Gaviria, 2008). Diffusion in the white matter of the brain is typically anisotropic due to the structure of an axon and its surrounding myelin sheath, such that diffusion along the neuronal fiber occurs readily, while diffusion perpendicular to the fiber is restricted (Medina and Gaviria, 2008; Wu and Lo, 2017). This diffusion anisotropy paints a picture of brain fiber tractography, organization, and architecture, by mapping white matter connections. Fractional anisotropy (FA) as well as axial diffusivity (AD), radial diffusivity (RD), and mean diffusivity (MD) can be used to characterize neuronal microenvironment changes due to pathological processes, such as axonal injury, neuroinflammation, or demyelination (Budde et al., 2007; Chang et al., 2017); whereas brain connectomes derived from graph theoretical analysis provide a systems view of the macroscopic brain network architecture (Watts and Strogatz, 1998; Bullmore and Sporns, 2009; Rubinov and Sporns, 2010; Estrada, 2011; Ingalhalikar et al., 2015). Graph theory defines brain regions and neuronal tracts between them as nodes and edges to quantitatively describe a variety of measures of network segregation and efficiency (Rubinov and Sporns, 2010). In turn, this allows a systems approach to study the architecture of the brain network. These tactics allow us to investigate local diffusion properties and the global brain network differences between ApoE KO and WT mice.

We performed DTI on age-matched WT and ApoE KO male mice to examine diffusion parameters and used graph theoretical analysis to evaluate differences in their neuronal fiber connectivity to delineate brain network architecture. Starting with the whole brain, we investigated differences in right and left hemisphere diffusivity and connectivity, for WT and ApoE KO separately. Though we saw no differences in diffusivity, we compared left and right hemisphere connectivity, without assigning any regions of interest (ROI) nor regions of avoidance (NOA). This served as an unbiased, data driven approach and allowed us to see what differences exist in brain network characteristics. Both the left and right ipsilateral connectivity revealed an increase in significantly different connections between the amygdala, hippocampus, caudate putamen and other brain regions in ApoE KO mice. From here we conducted a more specific pathway analysis on the amygdala, hippocampus and caudate putamen. Not only did these regions stand out with multiple differences in connectivity from our unbiased approach, but these brain regions are associated with known behavioral phenotypes of ApoE KO mice (D'Hooge and De Deyn, 2001; Li and Liberles, 2015). Next, we looked at contralateral connections (fiber seeds in the left hemisphere and their connections in the right hemisphere) of both the entire hemisphere and of the regions of interest established by the ipsilateral analysis. Mapping contralateral fibers would reveal any differences in long range fiber connectivity. Our study explores brain regions where ApoE affects neuronal network in a frequently used LOAD mouse model. Differences in these animals’ neuronal network add to our knowledge base of this animal model and suggests that ApoE plays a role in brain network development and organization, which could predispose brains to differential LOAD vulnerability or resiliency.

## 2 Methods

### 2.1 Animals and sample preparation

All animals received humane care in compliance with the NIH Office of Laboratory Animal Welfare (OLAW) guidelines. Animal protocols were approved by the University of Pittsburgh Institutional Animal Care and Use Committee (IACUC). Mice were provided with *ad libitum* water and chow.

ApoE KO breeding pairs (B6.129P2-Apoe^tm1Unc^/J) were obtained from Jackson Laboratory (JAX stock #002052) (Piedrahita et al., 1992). Homozygous male ApoE KO (*n* = 10) and WT littermates (*n* = 9) were included in the study. Once weaned on post-natal day p28, male littermates were housed with 2–4 males per cage and kept on a 12:12 h dark/light cycle until 22 weeks of age when their brains were harvested for DTI. Twenty-two weeks (5 months) of age is considered mature adult (Hagan, 2017; Sukoff Rizzo and Crawley, 2017). Animals were euthanized using 5% isoflurane for 10 min or until they did not respond to a foot prick with forceps. Cervical dislocation was then performed. A chest incision through the abdominal wall and ribs was made, exposing the heart. The brain was fixed by cardiac perfusion with 5 mL of 4% paraformaldehyde (PFA) in phosphate buffered saline (PBS) *via* cardiac puncture. The animal’s brain was carefully harvested, preserving its anatomy. The intact brain was then placed in 4% PFA for a minimum of 2 days at 4°C, followed by PBS at 4°C for 2 days, then 10% formalin. Two days prior to imaging, brain samples were rehydrated in PBS at 4°C. At the time of scanning, the fixed brain was removed from PBS and excess liquid was dried from the surface. The brain was then transferred to a custom MRI sample holder containing Fomblin-Y perfluoropolyether vacuum oil (Millipore Sigma, MW = 1800) to eliminate any tissue-to-air artifact.

### 2.2 3D isotropic diffusion tensor imaging acquisition

3D isotropic diffusion MRI images were acquired with spin echo diffusion preparation, field of view (FOV) = 4.0×1.1×1.1 cm^3^, acquisition matrix size 256x70x70, repetition time (TR) = 1,000 ms, echo time (TE) = 16.665 ms, diffusion gradient separation = 8 ms, diffusion gradient duration = 4 ms, 30 diffusion directions, b = 1,200 s/mm^2^ per direction, total acquisition time = 11h9m36s.

### 2.3 3D isotropic anatomical T_2_-weighted imaging

3D T_2_-weighted isotropic images were acquired with 3D Rapid Imaging with Relaxation Enhancement (RARE), a Fast Spin-Echo (FSE) sequence, with exactly the same geometry as the DTI scans, for anatomical registration in the same brain space with the following parameters: FOV = 4.0 × 1.1 × 1.1 cm^3^, matrix size 512 × 141 × 141, TR = 1,000 ms, TE = 12.00 ms, RARE factor = 8, effective TE = 48.00 ms, refocusing flip angle 180 deg., total acquisition time 5h33m12s.

### 2.4 Registration to Allen mouse template

Diffusion MRI was performed on two *ex vivo* mouse brains simultaneously. Thus, diffusion-weighted imaging (DWI) Nifti-volumes contained two mouse brains. In a first step, the 1st volume of the DWI data was used to detect each mouse brain *via* watershed segmentation and a manually guided segmentation approach. Based on this segmentation for multiple mouse brains, the DWI data of each animal was stored in separate 4D Nifti files.

Registration to the Allen brain template: The 3D-RARE data was registered to the Allen brain template as described previously (Koch et al., 2019) using ANTx2, a custom MATLAB toolbox. In short, the 3D-RARE volume was rigidly registered to the Allen brain template (Allen Institute for Brain Science, USA) and segmented into gray matter (GM), white matter (WM) and cerebrospinal fluid (CSF) tissue probability maps (TPMs) using the Unified Segmentation approach as implemented in SPM (Friston et al., 1994; Ashburner and Friston, 2005). For the segmentation task the tissue probability maps (TPMs) of Hikishima et al. (2017) served as priors for tissue classification. A weighted image was constructed using the tissue segments of the animal and the TPMs of the template, respectively. The weighted images were co-registered using affine and nonlinear B-spline transformation *via* Elastix package (Klein et al., 2010). The resulting parameter for forward transformation were stored to allow a subsequent image transformation from native animal space to Allen mouse template space. Parameter files for inverse transformation were also generated and stored to allow a subsequent image transformation from template space to native space (e.g., hemispheric brain mask). Using the files for inverse transformation, we transformed the template to the native, 1st volume of the DWI data to create the final brain segmentation mask.

A 72 region-based homolog atlas was generated by comprising or omitting regions of the 2017 Allen mouse brain atlas (Wang et al., 2020, see Table 1 for regions and abbreviations). The volume of this 72 region-based atlas, which is in register with the ABA in standard space, was transformed to native space *via* inverse transformation.

**Table 1:** Abbreviations for the atlas based parcellations are listed for the left (L) and right (R) hemisphere.

Region	Abbreviation
L corpus callosum	CC
L caudoputamen	CP
L anterior commissure olfactory limb	ACOL
L pallidum	PAL
L internal capsule	IntC
L thalamus	TH
L cerebellum	CB
L superior colliculus motor related	SUC
L ventricular systems	*VS*
L hypothalamus	HY
L inferior colliculus	IC
L periaqueductal gray	PAG
L isocortex	ICtx
L cortical amygdalar area	COA
L olfactory areas	OlfA
L pons	P
L midbrain reticular nucleus	RA
L nucleus accumbens	NA
L fimbria	F
L anterior cingulate area	ACA
L somatomotor areas	MO
L somatosensory areas	SS
L piriform area	PIR
L taenia tecta	TT
L accessory olfactory bulb glomerular layer	MOB_gl
L accessory olfactory bulb granular layer	MOB_gr
L retrohippocampal region	RHP
L entorhinal area	EC
L field CA1	CA1
L field CA3	CA3
L Dentate gyrus	DG
L field CA2	CA2
L accessory olfactory bulb mitral layer	MOB_mi
L striatum	STR
L midbrain	MB
L medulla	MY
R corpus callosum	RCC
R caudoputamen	RCP
R anterior commissure olfactory limb	RACOL
R pallidum	RPAL
R internal capsule	RIC
R thalamus	RTH
R cerebellum	RCB
R Superior colliculus motor related	RSUC
R ventricular systems	RVS
R hypothalamus	RHY
R Inferior colliculus	RInfC
R periaqueductal gray	RPAG
R isocortex	RICtx
R cortical amygdalar area	RCOA
R olfactory areas	ROlfA
R pons	RP
R midbrain reticular nucleus	RRN
R nucleus accumbens	RNA
R fimbria	RF
R anterior cingulate area	RACA
R somatomotor areas	RMO
R somatosensory areas	RSS
R piriform area	RPIR
R taenia tecta	RTT
R accessory olfactory bulb glomerular layer	RAOB_gl
R accessory olfactory bulb granular layer	RAOB_gr
R retrohippocampal region	RRHP
R entorhinal area	REC
R field CA1	RCA1
R field CA3	RCA3
R dentate gyrus	RDG
R field CA2	RCA2
R accessory olfactory bulb mitral layer	RAOB_mi
R striatum	RSTR
R midbrain	RMB
R medulla	RMY

### 2.5 Property and network analysis

#### 2.5.1 Diffusion parameters

The Allen brain atlas-based segmentation created 72 regions of interest (ROIs), 36 ROIs for each left or right hemisphere, and average diffusion parameters were calculated according to these ROIs by DSI studios. See https://sites.google.com/a/labsolver.org/dsi-studio/Manual/diffusion-mri-indices#TOC-DTI-based-metrics for more details. Definitions of diffusion-based metrics can be found in Table 2.

**Table 2:** Definitions of diffusion based metrics.

Diffusion parameter	Abbreviation	Definition
Fractional anisotropy	FA	Measures isotropic movement of water, in this case along axon bundles, 0 being that water diffuses freely and 1 being that water would diffuse entirely along one axis (Basser and Pierpaoli, 1996).
Axial diffusivity	AD	Measures diffusion along or parallel to axonal fibers (Winklewski et al., 2018).
Radial diffusivity	RD	measures diffusion perpendicular to axonal fibers (Winklewski et al., 2018)
Mean diffusivity	MD	Measures the average diffusion in all directions (Winklewski et al., 2018), also denoted as apparent diffusion coefficient (ADC).

#### 2.5.2 Tractography

Quantification of fiber tracking was completed using DSI studio^1^ (version 06/2018), with a minimum fiber tract length of 0, maximum fiber tract length of 300 mm, tracking algorithm RK4, angular threshold 0, and a total of 1,000,000 seeds calculated. Whole brain seeding for the entire brain as well as the left or right hemisphere and fiber tracking with the parameters above were conducted. We did not assign regions of interest but instead placed seeds over the entirety of the left hemisphere and reinstated the left hemisphere atlas afterwards to see where fiber tracts mapped to. Fiber tracts were then mapped to our 36 brain regions on the left hemisphere, so we were able to assess the amount of fiber tracts present in each region. This process was repeated by planting seeds in the right hemisphere and mapping the fibers to the right hemisphere. In addition to ipsilateral fiber tracking, we also looked at contralateral connections, to see if ApoE KO males would have differences in long-range axon patterning. In this case, we placed seeds in the left hemisphere and compared fiber numbers in the right hemisphere, and vice versa. When placing seeds in the left hemisphere to study long-range contralateral connections, ipsilateral connections will still track and are reflected in total fiber numbers and network parameters.

Fiber tracts for the hippocampus were generated by merging the left hemisphere’s Ammon’s Horn area 1 (CA1), Ammon’s Horn area 2 (CA2), Ammon’s Horn area 3 (CA3), and the dentate gyrus (DG). Fiber tracts for the amygdala were generated using the left hemisphere Cortical Amygdalar (COA) region. This was repeated in the right hemisphere by merging the RCA1, RCA2, RCA3, and RDG, and then the RCOA. Similarly, we assessed connectivity in the caudate putamen, and fiber tracts were generated using the left hemisphere caudate putamen region, then again with the right caudate putamen. Whole brain tract rendering was set to local index and either FA, MD, AD, or RD on a heat scale of 0–1 was specified in DSI studios.

Fiber tracts were assigned as direction for visualizing fiber tract directionality. Fiber tract color is indicative of fiber tract direction - red representing left–right fiber orientation, blue representing front-back fiber orientation, whereas green representation top-bottom orientation. Representative images of fiber tractography were generated using DSI studio’s (Mar 7 2020) simply for visual purposes. Any fiber tract analysis or quantification was conducted using only the 06/2018 version for consistency, but for visualizing whole-brain seed fiber tracts, the fiber tracts generated were visually improved in this version and employed for qualitative purposes.

#### 2.5.3 Adjacency matrix and connectogram

The hemisphere of the 72-region atlas-based parcellation used as the ROIs to create the adjacency matrices and connectograms depended on ipsilateral or contralateral analysis. For close-range fiber analysis, the hemisphere ipsilateral to where the seeds were placed of the 72-region atlas-based parcellation was used as the ROIs, and an adjacency matrix was calculated by using count of the connecting tracks in DSI studios after tractography. For long-range fiber analysis, the hemisphere contralateral to where the seeds were placed was used for calculation of the count of connecting tracts. The graph theory extraction threshold for both ipsilateral and contralateral was 0.001. In contralateral analysis, heat maps will display all 72 × 72 regions. Chord diagrams of adjacency matrices were generated using Circos table viewer,^2^ where row and column size were matched creating ribbons that are to scale with the number of connections between two regions of interest, each color representing a different region. Graphs are undirected. Chord diagrams of contralateral data display cross hemisphere connections, either 36 × 36 left seeds to map tracks to right regions and vice versa, to avoid redundancy. Representative ball and stick plots are 3D graph visualizations in which balls represent nodes and sticks represent edges. The size of the ball corresponds to the number of connections in a brain region, and the width of the sticks is proportional in size to the number of edges between nodes. As such, these are visual representations of our network parameters.

#### 2.5.4 Network topography

Using the same tractography that made the adjacency matrices, graph theoretical analysis was calculated by DSI studio for various network parameters. The graph theory extraction threshold was 0.001. These network parameters included measures of efficiency, global efficiency and small worldness, and measures of segregation, local efficiency and clustering coefficient. The following graph theory definitions can be found in more detail in Table 3 (Rubinov and Sporns, 2010; Ingalhalikar et al., 2015; Scharwächter et al., 2022).

**Table 3:** Definitions of network parameters.

Network parameter	Definition
Global efficiency	Quantifies connectedness and potential communication between regions by taking the inverse of the characteristic path length
Local efficiency	Describes connectedness around a node, or the shortest path length between two regions and is considered a measure of segregated information
Clustering coefficient	The proportion of the number of actual connections between nodes and their next closest neighbors and the number of possible connections between nodes and their next closest neighbors
Small worldness	The degree of high clustering paired with short path lengths

**Figure 1:**
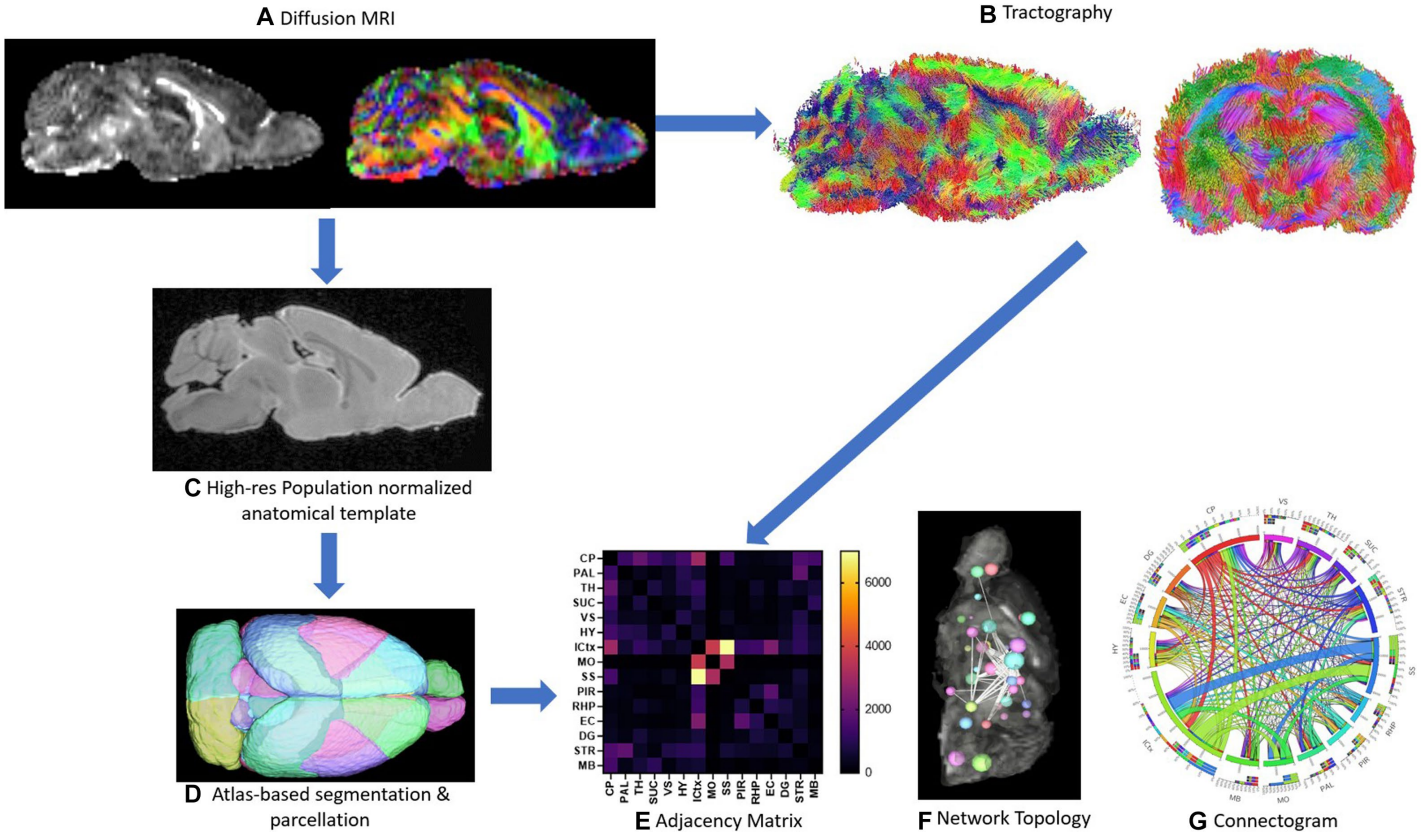
Schematic of methods is depicted. Representative sagittal slices of rodent diffusion MRI scans with voxel contrast assigned as FA (left) and color (right) are shown **(A)**. Tractography of the whole brain without assigning an ROI or ROA is demonstrated from both the sagittal and axial viewpoints **(B)**. The high-resolution population normalized anatomical template and atlas-based segmentation and parcellation of the 72-brain region atlas are demonstrated **(C,D)**. A sample adjacency matrix and connectogram of connections mapped to brain regions **(E,G)** as well as a representative ball-and-stick model of network topology **(F)** are demonstrations of subsequent network analysis conducted steps 1A–1D **(E-G)**.

The process from imaging to network topology and connectogram are depicted in Figure 1. Characterizing neuronal networks into network measures through graph theory describes the physiological aspect of information processing, quantifying structure and function (Bullmore and Sporns, 2009). Network measures in DSI studio follow the implementation of the brain connectivity toolbox. Graph theoretical analysis treats brain connections like a graph, so its topology can be quantitatively described by network parameters. We graph weighted measures, such that the connectivity matrix will be normalized so the maximum value of the matrix is one.

### 2.6 Statistical analysis

Statistical comparisons of volume, FA, AD, MD, and RD were assessed by multiple unpaired t-tests, with correction for multiple comparisons, two-stage step-up (Benjamini, Krieger, and Yekutieli) with desired false discover rate (FDR) at 5.00%.

Differences between WT and KO brain connectivities were performed by comparing the distribution of fiber tracts in between all 36(36–1)/2 = 630 pairs of brain regions. We excluded pairs where >50% of their observations were 0 and, to alleviate skewing and make the data more amenable to linear models, log-transformed the data after adding a pseudo count of 1 to avoid taking the log of zero. T-tests with n_WT_ + n_KO_ – 2 = 17 degrees of freedom were used to compare WT and KO pairs and resulting *p*-values were adjusted using the Benjamini Hochberg procedure to adjust for multiple testing by controlling the FDR at 5% (Benjamini and Hochberg, 1995). These computations were performed in Rv4.1.2.

Statistical comparisons of network parameters and total connections were evaluated using unpaired, two-tailed t-tests, with confidence level 95%. Definition of statistical significance: *p* < 0.05.

## 3 Results

### 3.1 Left vs. right hemisphere diffusivity analysis

**Figure 2:**
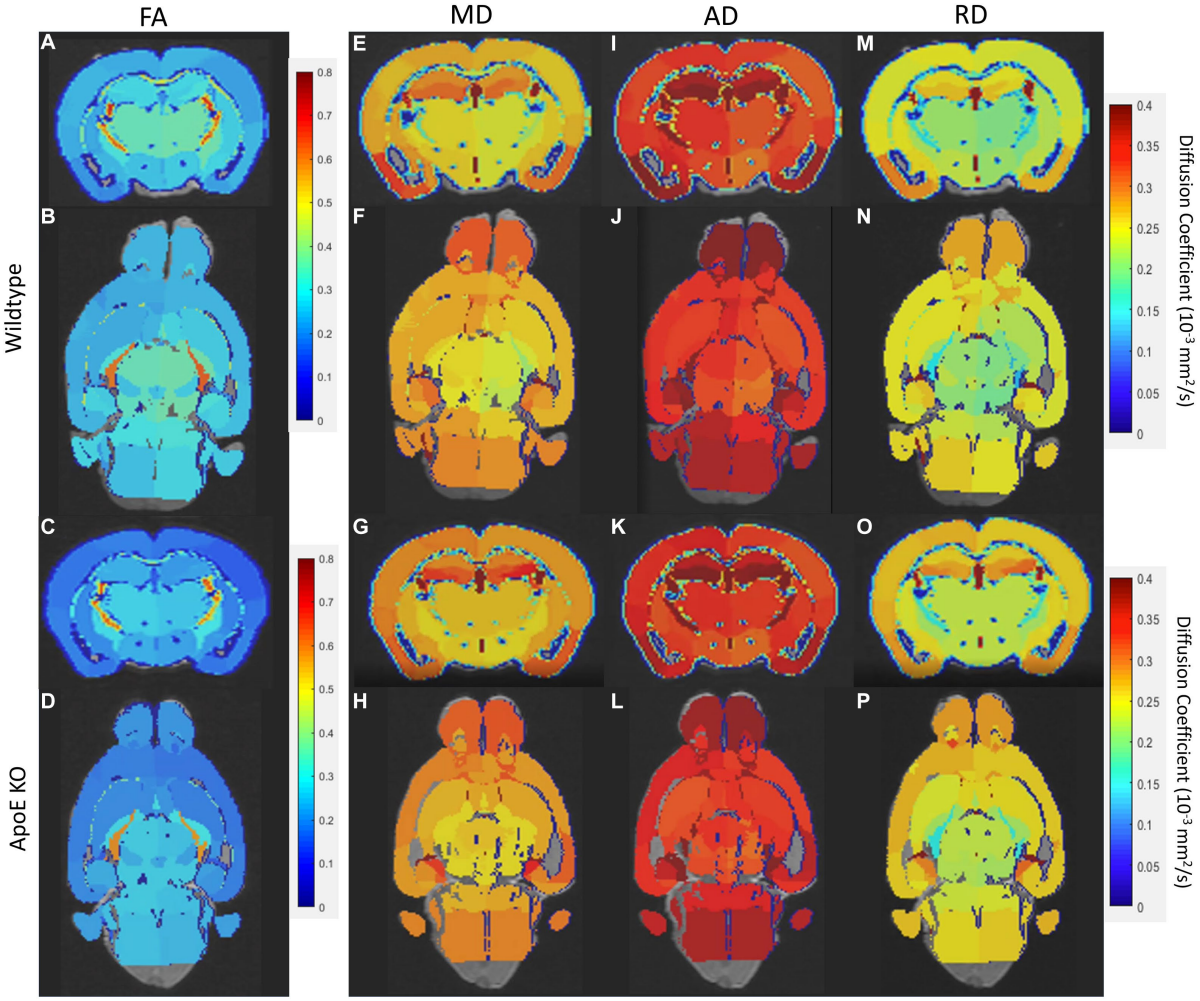
Representative images of whole brain of both the left and right hemisphere have been rendered on a scale of 0–0.4 for average fractional anisotropy (FA), medial diffusivity (MD), axial diffusivity (AD), and RD (radial diffusivity) per ROI. General patterning of all diffusion parameters appears the same in both the left and right hemisphere.

We first examined if left and right hemispheres are different in measures of volume and diffusivity. Volume and diffusion parameters, such as FA, RD, AD and MD, per ROI per hemisphere were averaged for both WT and ApoE KO, in order to determine differences between the right and left hemisphere of either strain. According to two-sample unpaired t-test, no regions were significantly different after adjusting for FDR at 5% in the left or right for both ApoE KO and WT (Supplementary Tables S1, S2). *p*-values between left and right ROIs are all greater than 0.05. Representative images of both WT and ApoE KO average FA (2A, I and B, J respectively), MD (2C, K and D, L), AD (2E, M and F, N) and RD (2G, O and H, P) per ROI are shown on a color scale of diffusion coefficient (Figure 2). Qualitatively, axial views of both left and right hemispheres also show similar patterning between the two for both WT and ApoE KO. There are no detectable differences between the left and right hemispheres for the parameters examined.

#### 3.1.1 ApoE KO vs. WT ipsilateral hemisphere analysis

Next, we conducted an unbiased comparison between ApoE KO and WT for the entire left hemisphere ipsilateral connectivity followed by the entire right hemisphere ipsilateral connectivity. Since we saw little physical or volumetric differences, comparing average tractography between WT and ApoE KO groups could illustrate significant differences in specific areas of the brain. A unilateral analysis of the left hemisphere led to differences in fiber tract orientation (representative images 3A and C, top). These left fiber tracts were then mapped to our 36 brain regions on the left hemisphere (representative images 3A and 3C, middle) so we were able to assess the amount of fiber tracts present in each region, as well as visualize the 3D graphs *via* ball and stick plots (3A and 3C, bottom). From our whole brain analysis, we created an average connectogram plot (WT 3B and ApoE KO 3D, top) from the average adjacency matrix (WT 3B and ApoE KO 3D, bottom).

We compared average numbers of fiber tracts in the left hemisphere in both WT and ApoE KO and found there were significantly more fibers in ApoE KO than WT (Table 4). While the average connectogram plots (3B and 3D) demonstrate that connectivity patterns are typically similar between WT and ApoE KO, as in the ribbons representing connections between two regions exist in a similar pattern, differences in connectivity amounts are discernable from the heatmaps of raw fiber tract numbers.

**Table 4:** Average number of connections for each area of analysis are shown.

	WT	ApoE	*P-*value
Left hemisphere ipsilateral	499,715 ± 12,109	512,639 ± 13,637	0.042
Right hemisphere ipsilateral	525,555 ± 21,387	523,448 ± 24,227	0.84
Left hemisphere contralateral	654,260 ± 115,659	657,827 ± 98,715	0.94
Right hemisphere contralateral	685,326 ± 109,612	673,759 ± 66,514	0.78
Left hippocampus ipsilateral	1.16 ± 0.17*10^5^	1.08 ± 0.17*10^5^	0.356
Right hippocampus ipsilateral	124,966 ± 24,252	116,930 ± 19,822	0.44
Left hippocampus contralateral	143,272 ± 22,555	140,201 ± 39,565	0.84
Right hippocampus contralateral	143,194 ± 31,387	142,295 ± 38,426	0.96
Left amygdala ipsilateral	2.56 ± 0.87*10^4^	2.08 ± 1.3*10^4^	0.3622
Right amygdala ipsilateral	28,883 ± 9,387	19,881 ± 5,781	0.021
Left amygdala contralateral	17,287 ± 9,898	11,090 ± 6,398	0.12
Right amygdala contralateral	21,813 ± 7,052	18,062 ± 10,973	0.39
Left caudate putamen ipsilateral	88,226 ± 20,939	83,502 ± 19,580	0.62
Right caudate putamen ipsilateral	85,319 ± 15,163	75,513 ± 10,883	0.12
Left caudate putamen contralateral	180,506 ± 23,124	183,104 ± 42.176	0.87
Right caudate putamen contralateral	180,202 ± 28,505	169,544 ± 38,151	0.5

**Figure 3:**
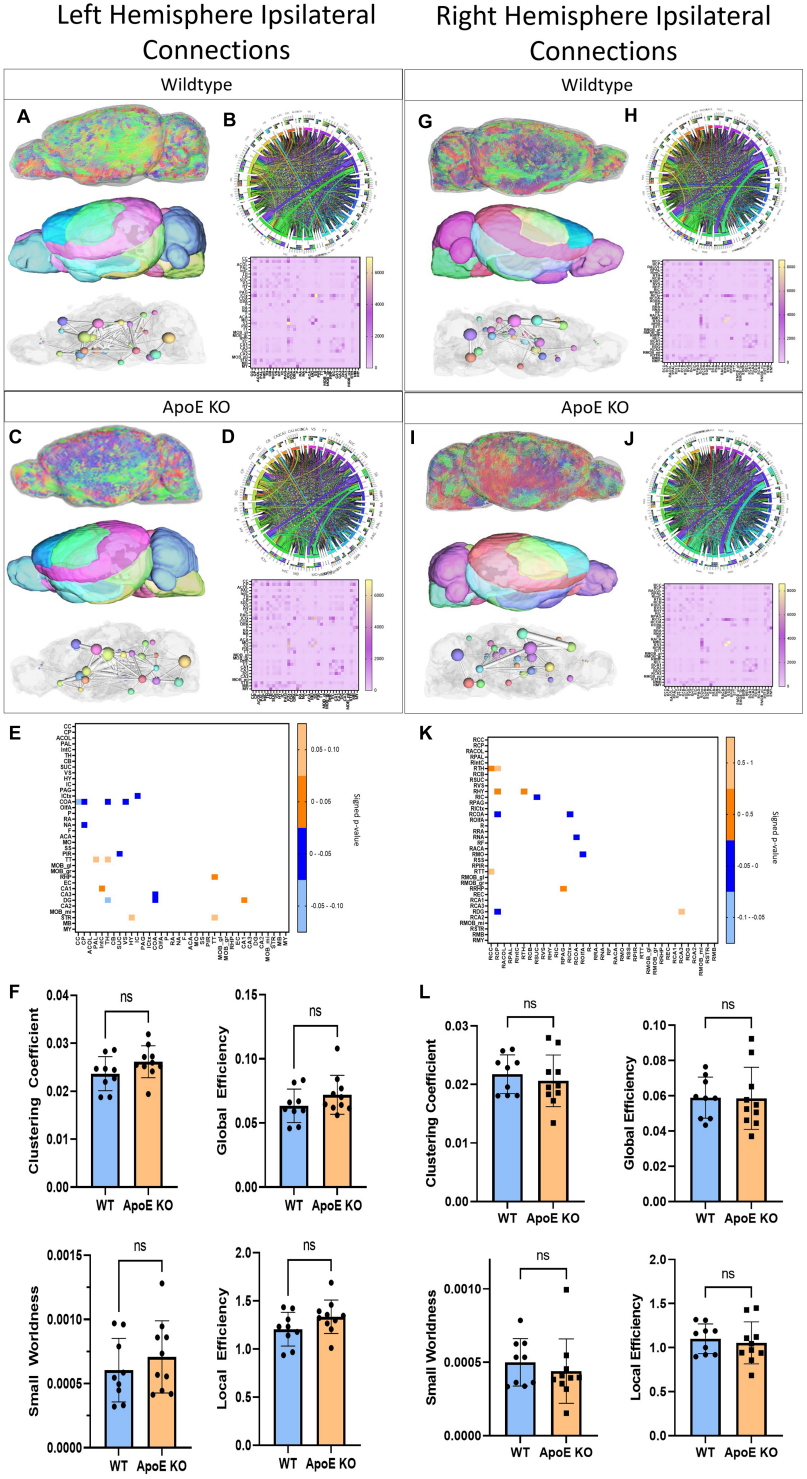
Left Hemisphere ipsilateral fiber tracking results are depicted for WT **(A,B)** and ApoE KO **(C,D)**. To compare connectomes, an adjacency matrix of signed FDR adjusted *p*-values shows connections between regions where ApoE KO > WT (0.1–0.05 light orange, 0.05–0.0 dark orange) and connections between regions where WT > ApoE KO (0.0- –0.05 dark blue, −0.05- –0.1 light blue) **(E)**. Average network parameters **(F)**, clustering coefficient, small worldness, global efficiency and local efficiency are compared for WT and ApoE KO, *t*-test was used to determine significance. Right hemisphere ipsilateral fiber tracking results are depicted for WT **(G,H)** and ApoE KO **(I,J)**, an adjacency matrix comparing connections **(K)** and network parameters **(L)** are graphed.

Our analysis showed that the group averaged ApoE KO brains had significantly higher numbers of fiber tracts, so we then conducted regional analysis to pinpoint exactly which connections between regions were greater than WT. Using the adjacency matrices, we were able to compare the number of fiber tracts between one region and every other region, for all 36 ROIs. This found that only 2 connecting regions had significantly higher numbers of connections in ApoE KO than WT (see Table 5 for significant connecting regions). This statistical analysis revealed, however, that 9 connecting regions were significantly lower in ApoE KO mice than WT (Table 5 and visualized in Figure 3E).

**Table 5:** Significant differences in connection regions for each area where fibers were seeded are presented.

Area of interest	Connecting regions	FDR adjusted *p*-value
Left hemisphere ipsilateral			Significantly higher in ApoE KO		RHP –TT	0.046	DG – CA1	0.041	Significantly higher in WT		COA-CP	0.041	COA-TH	0.013	COA-*VS*	0.004	COA-CA3	0.01	COA-DG	0.013	ICtx-IC	0.041	NA-CP	0.019	PIR-SUC	0.013	CA1-IntC	0.00043
	Right hemipshere ipsilateral			Significantly higher in ApoE KO		RTH-RCC	0.024	RHY-RCP	0.024	RHY-RTH	0.003	RRHP-RPAG	0.042	Significantly higher in WT		RCOA-CP	0.026	RCOA-RICtx	0.019	RinfC-RSUC	0.024	RNA-RolfA	0.019	RMO-RolfA	0.007	RDG-RCP	0.024
		Left hippocampus ipsilateral			Significantly higher in ApoE KO		CC –TT	0.02	DG – CA1	0.034	Significantly higher in WT		TH-CP	0.048	PIR-CP	0.019	IC-CA1	0.009	COA-CA3	0.009	COA-DG	0.009	COA-TH	0.009	*VS*-COA	0.009	PIR-ICTX	0.05
			Left amygdala ipsilateral			Significantly higher in WT		COA-CC	0.015	COA-CP	0.015		COA-TH	0.001	COA-SUC	0.031	COA-*VS*	0.001	COA-HY	0.031	COA-SS	0.031	COA-CA3	0.004	COA-DG	0.015	COA-MB	0.044
				Right amydala ipsilateral			Significantly higher in ApoE KO		RPIR-RCOA	0.018	RPIR-REC		0.014	RPIR-RSTR	0.014	Significantly higher in WT		RCOA-RCC	0.033	RCOA-RCP	0.014	RCOA-RVS	0.005	RCOA-RICtx	0	ROlfA-RTH	0.032	ROlfA-RVS	0.049	ROlfA-RICtx	0.014	RCOA-RSS	0.007	RPIR-RICtx	0.018	RCOA-RCA1	0.015	RCOA-RDG	0.014	RCOA-RMB	0.05	ROlfA-RDG	0.015	RICtx-RSTR	0.003
					Left amygdala contralateral			Significantly higher in WT		COA-RCC	0.026		COA-RSUC	0.036	COA-RVS	0.036	COA-RPAG	0.026	COA-RICtx	0.039	COA-RRHP	0.026	COA-RCA3	0.036
						Left caudate putamen ipsilateral			Significantly higher in WT		COA-DG		0.013
			Right caudate putamen ipsilateral						Significantly higher in WT		RCOA-RPAL		0.001	RCOA-RHY	<0.00001	RCOA-RICtx	0.001	RCOA-ROlfA	0.021	RCOA-RSTR	0.023

Next, we compared network measures between ApoE KO and WT left hemisphere connectivity. We found no significant differences in clustering coefficient, small worldness, global or local efficiency (Figure 3F).

Overall, while we found that while ApoE KO averaged significantly higher numbers of connections in the ipsilateral left hemisphere connections, this greater connectivity was only significantly different between two connecting pairs and was not associated with significant differences in network measures. In the 9 connections where ApoE KO connection numbers were significantly lower than WT, 3 of the 9 connection pairs involved the hippocampus, and 5 involved subregions of the amygdala.

In the right hemisphere ipsilateral analysis, we similarly compared average numbers of fiber tracts and found no significant difference between WT and ApoE KO (Table 4). Representative fiber tracts, right hemisphere ROIs, and ball-and-stick plots of right hemisphere connectivity demonstrate visual connectivity (Figures 3G,I), and average connectogram plots and heatmaps of raw fiber tract numbers (Figures 3H,J) demonstrate again that connectivity patterns are typically similar between WT and ApoE KO.

Using the adjacency matrixes, we compared the number of fiber tracts between one region and every other region. This found significantly higher numbers of connections in ApoE KO than WT between 4 regions (Table 4). This analysis also revealed 6 regions were significantly lower in ApoE KO vs. WT (Table 5). While these results returned slight differences than the left ipsilateral hemisphere connections, the highest number of significantly different connecting regions are still found in the hippocampus, amygdala, and caudate putamen.

When comparing right ipsilateral weighted network measures, we found, similar to the left ipsilateral analysis, that there are no significant differences between WT and ApoE KO in clustering coefficient, small worldness, local efficiency, and global efficiency (Figure 3L).

#### 3.1.2 ApoE KO vs. WT contralateral hemisphere analysis

**Figure 4:**
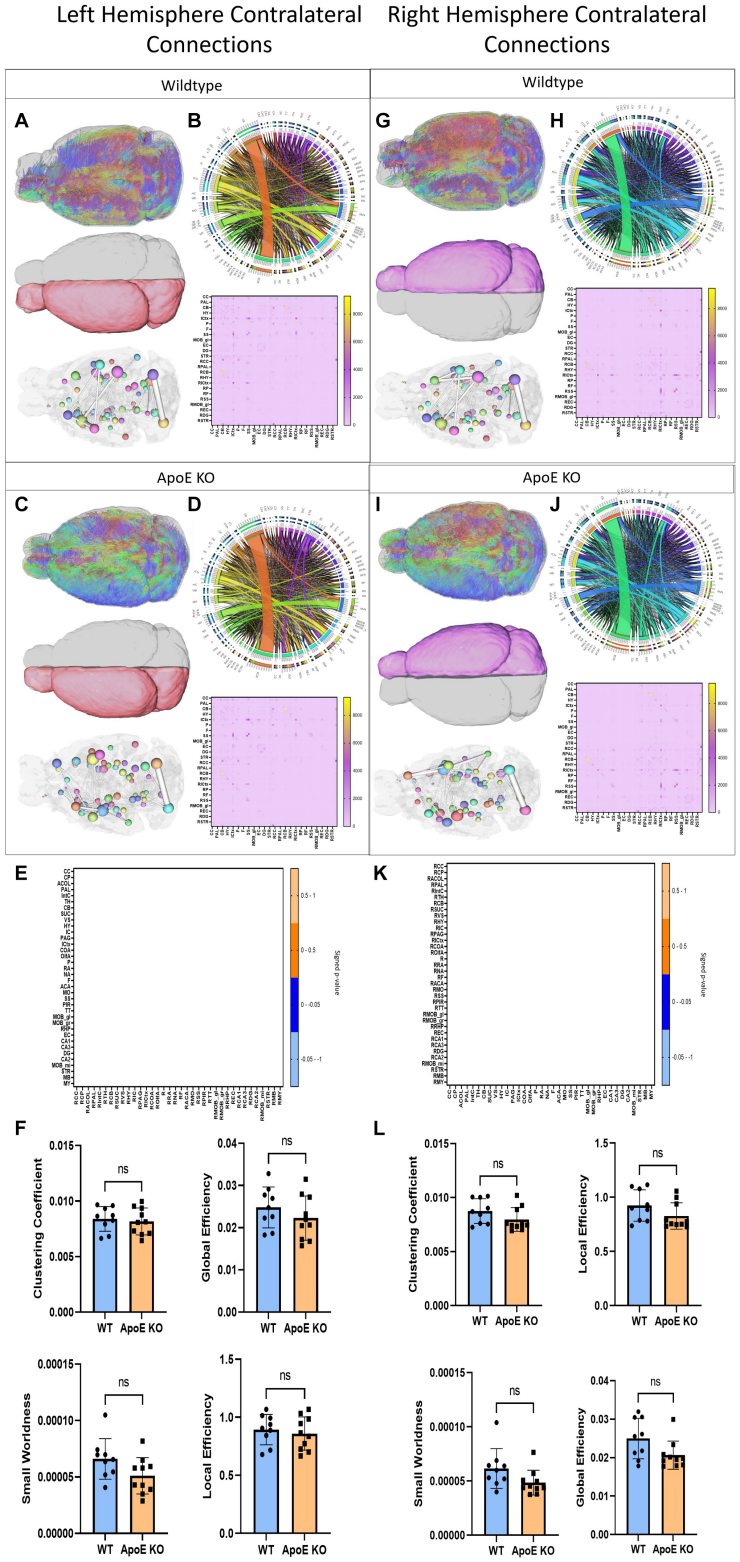
Left Hemisphere contralateral fiber tracking results are depicted for WT **(A,B)** and ApoE KO **(C,D)**. To compare connectomes, an adjacency matrix of signed FDR adjusted *p*-values shows connections between regions where ApoE KO > WT (0.1 -0.05 light orange, 0.05 -0.0 dark orange) and connections between regions where WT > ApoE KO (0.0- -0.05 dark blue, −0.05- -0.1 light blue) **(E)**. Average network parameters **(F)**, clustering coefficient, small worldness, global efficiency and local efficiency are compared for WT and ApoE KO, *t*-test was used to determine significance. Right hemisphere ipsilateral fiber tracking results are depicted for WT **(G,H)** and ApoE KO **(I,J)**, an adjacency matrix comparing connections **(K)** and network parameters **(L)** are graphed.

To explore ApoE’s effects on long-range neuronal development, we planted seeds in the left hemisphere and mapped their connections to the right hemisphere. Representative fiber tracts, ROIs and ball and stick models demonstrate contralateral connectivity (Figures 4A,C). We did not find a significant difference in total average fiber tract between ApoE KO and WT (Table 4). When comparing the number of fiber tracts between one region and every other region in the adjacency matrices (Figures 4B,D), we found no significant differences in number of connections between any two regions (Table 5 and Figure 4E). We compared ApoE KO and WT left hemisphere network parameters, including contralateral connections and found no significant differences, clustering coefficient (WT: 0.0084 ± 0.0011, ApoE KO: 0.0082 ± 0.0012, *p* = 0.67), small worldness (WT: 6.61E-05 ± 1.8E-05, ApoE KO: 5.10E-05 ± 1.61E-05, *p* = 0.071), local efficiency (WT: 0.89 ± 0.13, ApoE KO: 0.86 ± 0.14, *p* = 0.60), and global efficiency (WT: 0.023 ± 0.0048, ApoE KO: 0.022 ± 0.0052, *p* = 0.29) (Figure 3F).

In a similar fashion, we planted seeds in the right hemisphere and mapped their connections and compared network measures in the left hemisphere (Figures 4G,I). Again, we did not find a significant difference in total average fiber tract between ApoE KO and WT (Table 4) Here, we also found no significant differences in number of connections between any two regions from the adjacency matrices (Table 5 and Figure 4K). We compared ApoE KO and WT network parameters and none were significantly different, including clustering coefficient (WT: 0.0097 ± 0.0030, ApoE KO: 0.0091 ± 0.0037, *p* = 0.68), small worldness (WT: 3.20E-05 ± 1.35E-05, ApoE KO: 2.98E-05 ± 2.08E-05, *p* = 0.79), global efficiency, though it approached significance, (WT: 0.025 ± 0.0052, ApoE KO: 0.021 ± 0.0037, *p* = 0.0509), and local efficiency (WT: 0.84 ± 0.22, ApoE KO: 0.77 ± 0.30, *p* = 0.58) (Figure 4L).

#### 3.1.3 ApoE KO vs. WT left and right ipsilateral hippocampus analysis

The 4 regions in the ipsilateral fiber tract analysis with significantly fewer average connections to subregions of the hippocampus (COA-CA3, COA-DG, CA1-IntC, RDG-RCP) in ApoE KO compared to WT, as well as the ipsilateral connections that were higher in ApoE KO than WT (DG-CA1 and RDG-RCA3) led us to wonder what fiber tract organization looked like when fiber tracts running through the hippocampus were isolated. Using automated segmentations and the same tracking parameters as the ipsilateral hemisphere analysis, we placed 1,000,000 fiber tract seeds only in the left or right hippocampus (CA1, CA2, CA3, and the dentate gyrus) and mapped their connections into the rest of the ipsilateral hemisphere. In the left hippocampus, an outline of the ROI is depicted for WT and ApoE KO respectively, and these ipsilateral hippocampus fiber tracts were similarly mapped to our 36 brain regions on the left hemisphere (Supplementary Figures S1A,C) so we can assess the amount of fiber tracts present in each region. In the same manner as the ipsilateral hemisphere, we used graph theoretical analysis to describe the organization of these fiber tracts. Representative ball and stick plots are 3D graph visualizations (Supplementary Figures S1B,D), in which balls represent nodes and sticks represent edges.

We found no significant difference in the average number of tracts derived from the left hippocampus (Table 4). To explore this, we again compared the number of fiber tracts between one region and every other region, for all 36 ROIs, this time using hippocampus fiber tracts rather than left hemisphere fiber tracts (see Supplementary Figure S1 for details). This found that only 2 connecting regions had significantly higher numbers of connections in ApoE KO than WT. On the other hand, 8 connecting regions were significantly lower in ApoE KO mice than WT (Table 5). Many of the significantly lower connections in this case also involve the amygdala and hippocampus, indicating there is a deficit in connectivity between these regions and the rest of the brain.

**Figure 5:**
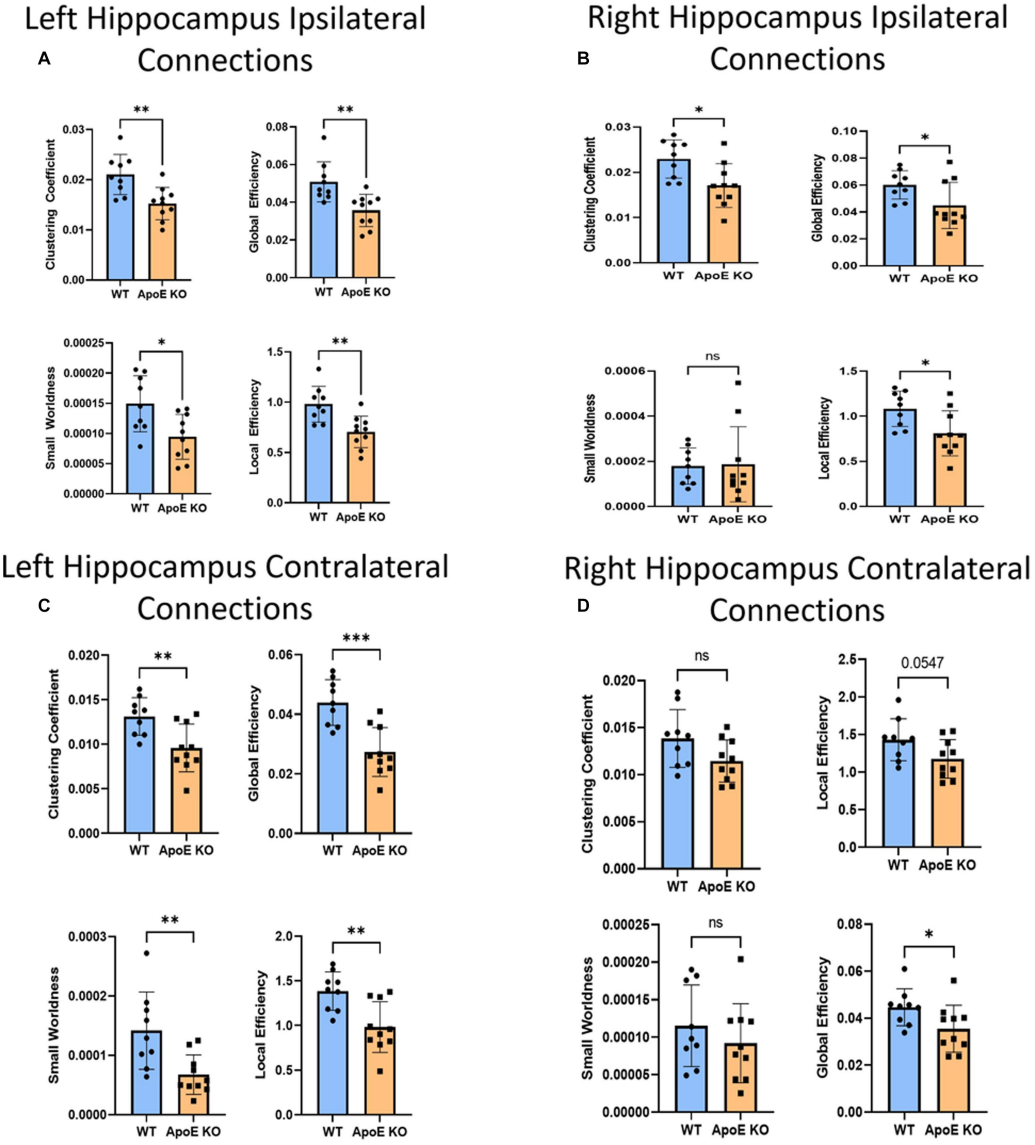
Average network parameters derived from hippocampus-seeded fiber tracking for left ipsilateral (**A**, top right: clustering coefficient, bottom right: small worldness, top left: global efficiency and bottom left: local efficiency), right ipsilateral **(B)**, left contralateral **(C)**, and right contralateral **(D)** are compared for WT and ApoE KO, *t*-test was used to determine significance. * signifies *p* ≤ 0.05; ** signifies *p* ≤ 0.01; *** signifies *p* ≤ 0.001.

This time, when comparing ApoE KO and WT network parameters, we found WT was significantly greater than ApoE KO in 4 parameters according to student’s two-sample t-test: Clustering coefficient (WT: 0.021 ± 0.0040, ApoE KO: 0.015 ± 0.0032, *p* = 0.0028), small worldness (WT: 0.00015 ± 4.62E-05, ApoE KO: 9.45E-05 ± 3.71E-05, *p* = 0.011), local efficiency (WT: 0.98 ± 0.18, ApoE KO: 0.71 ± 0.16, *p* = 0.0023), and global efficiency (WT: 0.051 ± 0.011, ApoE KO: 0.036 ± 0.00085, *p* = 0.0031) (Figure 5A).

WT and ApoE KO male’s right hippocampus derived fiber tracts were not significantly different (Table 4). See Supplementary Figure S2 for more details. When comparing fiber tracts between regions ipsilateral to the right hippocampus, we only found one pair of connection regions were significantly lower in WT compared to ApoE KO (Table 5 and Supplementary Figure 1J). We did, however, find significant differences in network parameters. We found that WT was significantly greater in 3 network parameters (Figure 5B), clustering coefficient (WT: 0.023 ± 0.0042, ApoE KO: 0.017 ± 0.0048, *p* = 0.012), global efficiency (WT: 0.060 ± 0.011, ApoE KO: 0.045 ± 0.017, *p* = 0.034), and local efficiency (WT: 1.08 ± 0.20, ApoE KO: 0.81 ± 0.25, *p* = 0.018). Small worldness, however, was not significantly different (WT: 0.00018 ± 8.04E-05, ApoE KO: 0.00019 ± 1.6E-04, *p* = 0.90).

In the hippocampus fiber tracts specifically, we found that while overall fiber tract numbers were not significantly different between ApoE KO and WT in either the left or right ipsilateral connections, 8 connecting regions were significantly lower in ApoE KO than WT, all from the left ipsilateral hippocampal connections, and 3 were significantly higher, many of these regions again involving hippocampus and amygdala. Further, we found that network measures, including measures of both integration and segregation which are keys to an efficient network, were significantly lower in ApoE KO than WT in both hemispheres.

#### 3.1.4 ApoE KO vs. WT contralateral hippocampus analysis

In order to explore ApoE’s effects on long-range neuronal development in the hippocampus, we planted seeds in the left hippocampus and also mapped their connections to the right hemisphere (see Supplementary Figure S2). We found no significant difference in the average number of fiber tracts (Table 4). There were also no significant differences in contralateral fiber numbers between any two regions when comparing ApoE KO and WT adjacency matrices (Table 5). We compared ApoE KO and WT network parameters and found WT was significantly higher in 4 different parameters (Figure 5C) clustering coefficient (WT: 0.013 ± 0.0021, ApoE KO: 0.0096 ± 0.0027, *p* = 0.0056), small worldness (WT: 0.00014 ± 6.51E-05, ApoE KO: 6.73E-05 ± 3.32E-05, *p* = 0.0055), local efficiency (WT: 1.39 ± 0.22, ApoE KO: 0.98 ± 0.28, *p* = 0.0030), and global efficiency (WT: 0.044 ± 0.0077, ApoE KO: 0.027 ± 0.0082, *p* = 0.0003).

When exploring ApoE’s effects on long-range neuronal development in the right hippocampus, we planted seeds in the right hippocampus and mapped their connections to the left hemisphere (Supplementary Figure S2). There were, again, no significant differences in average fiber numbers and no significant differences in contralateral fiber numbers between any two regions when comparing ApoE KO and WT adjacency matrices (Table 5). We compared ApoE KO and WT network parameters and found that clustering coefficient (WT: 0.014 ± 0.0031, ApoE KO: 0.011 ± 0.0022, *p* = 0.067), small worldness (WT: 0.00012 ± 5.43E-05, ApoE KO: 9.19E-05 ± 5.27E-05, *p* = 0.35), and local efficiency were not significantly different, though local efficiency was approaching significance (WT: 1.43 ± 0.28, ApoE KO: 1.18 ± 0.26, *p* = 0.055). Global efficiency, however, was significantly different (WT: 0.045 ± 0.0079, ApoE KO: 0.036 ± 0.010, *p* = 0.0427) (Figure 5D).

#### 3.1.5 ApoE KO vs. WT ipsilateral amygdala analysis

Our left hemisphere approach revealed significant deficiencies in 5 areas and our right hemisphere analysis revealed significant deficiencies in 3 areas connecting with the amygdala, which again led us to wonder what fiber tract organization looked like when isolating the amygdala. We repeated the same process as with the hippocampus, instead using the amygdala as our ROI, again placing 1,000,000 seeds in the region. See Supplementary Figure S3 for more details.

**Figure 6:**
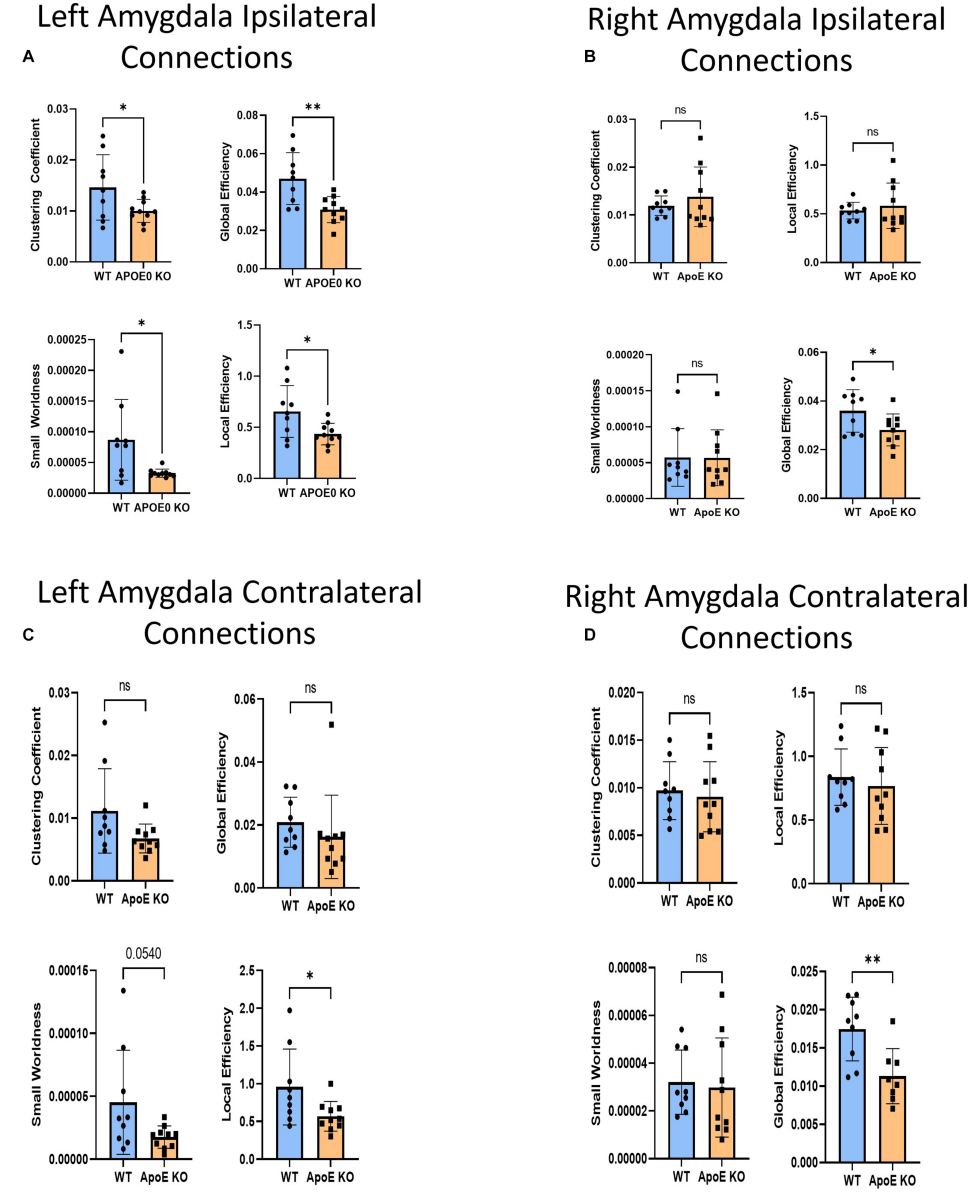
Average network parameters derived from amygdala-seeded fiber tracking for left ipsilateral (**A**, top right: clustering coefficient, bottom right: small worldness, top left: global efficiency and bottom left: local efficiency), right ipsilateral **(B)**, left contralateral **(C)**, and right contralateral **(D)** are compared for WT and ApoE KO, *t-*test was used to determine significance.

There was no significant difference in average fiber tract amount between WT and ApoE KO, despite noticeably different patterns in connectograms (Table 4 and Supplementary Figure S3). We then compared the number of amygdala fiber tracts between one region and every other region, for all 36 ROIs. This time, we found 10 regions of interest all involving the left amygdala had significantly lower numbers of connections in ApoE KO than in WT (Table 5). All of the significantly lower connections in this case involve the amygdala, alluding to a deficit in amygdala connectivity. Further, network parameters pertaining to amygdala fiber tract organization and efficiency were significantly lower in ApoE KO than WT according to student’s t-test: clustering coefficient (WT: 0.015 ± 0.0064, ApoE KO: 0.010 ± 0.0023, *p* = 0.048), small worldness (WT: 8.7E-05 ± 6.58E-05, ApoE KO: 3.27E-05 ± 6.67E-06, *p* = 0.019), local efficiency (WT: 0.65 ± 0.25, ApoE KO: 0.43 ± 0.11, *p* = 0.022), and global efficiency (WT: 0.047 ± 0.014, ApoE KO: 0.031 ± 0.00068, *p* = 0.0039) (Figure 6A).

We conducted fiber tracking and network analysis for right ipsilateral amygdala connections (Supplementary Figure S3). The average number of fiber tracts derived from WT right amygdala was significantly higher than that of ApoE KO (Table 4). While ApoE KO mice have significantly fewer total fibers, we found that three pairs of connecting regions had significantly higher numbers of fibers than WT. On the other hand, we found that 14 regions had significantly lower numbers of connections in ApoE KO than WT (Table 5). When comparing ApoE KO and WT network parameters, we found that WT was not significantly different in 3 network parameters, clustering coefficient (WT: 0.012 ± 0.0021, ApoE KO: 0.014 ± 0.0062, *p* = 0.40), small worldness (WT: 5.7E-05 ± 4.0E-05, ApoE KO: 5.7E-05 ± 3.9E-05, *p* = 0.98), local efficiency (WT: 0.53 ± 0.086, ApoE KO: 0.58 ± 0.23, *p* = 0.56). Global efficiency was, however, significantly different (WT: 0.036 ± 0.0087, ApoE KO: 0.028 ± 0.0066, *p* = 0.040) (Figure 6B).

#### 3.1.6 ApoE KO vs. WT contralateral amygdala analysis

In order to explore ApoE’s effects on long-range neuronal development in the amygdala-seeded connectome, we planted seeds in the left COA and right COA and both fiber tract counts and network parameters when mapped to the right hemisphere (Supplementary Figure S4). There was no significant difference in fiber tract amount between WT and ApoE KO (Table 4). We found that seven pairs of connecting regions had significantly higher numbers of fibers in WT than in ApoE KO (Table 5). Though significant differences were found in contralateral left amygdala fiber analysis, nearly all the pairwise connection measurements for the bilateral COA were 0. As such, any non-NA adjusted value of *p*s falling below 0.05 may be spurious. We compared ApoE KO and WT network parameters, and found that WT was significantly higher in 1 network parameters, and nearing significance in 2 others (Figure 6C), clustering coefficient (WT: 0.011 ± 0.0067, ApoE KO: 0.0068 ± 0.0023, *p* = 0.0677), small worldness (WT: 4.52E-05 ± 4.14E-05, ApoE KO: 1.75E-05 ± 8.87E-06, *p* = 0.054), local efficiency (WT: 0.96 ± 0.50, ApoE KO: 0.57 ± 0.20, *p* = 0.037), while global efficiency was not significantly different (WT: 0.021 ± 0.0080, ApoE KO: 0.016 ± 0.0045, *p* = 0.37).

In the right contralateral analysis, WT males average number of connections and ApoE KO average number of connections were not significantly different (Table 4). In this hemisphere, however, we found no significant differences in connections between regions. We compared ApoE KO and WT network parameters and none were significantly different, in clustering coefficient (WT: 0.0097 ± 0.0030, ApoE KO: 0.0091 ± 0.0037, *p* = 0.68), small worldness (WT: 3.20E-05 ± 1.35E-05, ApoE KO: 2.98E-05 ± 2.08E-05, *p* = 0.79), global efficiency (WT: 0.017 ± 0.0042, ApoE KO: 0.019 ± 0.0166, *p* = 0.82), and local efficiency (WT: 0.84 ± 0.22, ApoE KO: 0.77 ± 0.30, *p* = 0.58) (Figure 6D).

#### 3.1.7 ApoE KO vs. WT ipsilateral caudate putamen analysis

**Figure 7:**
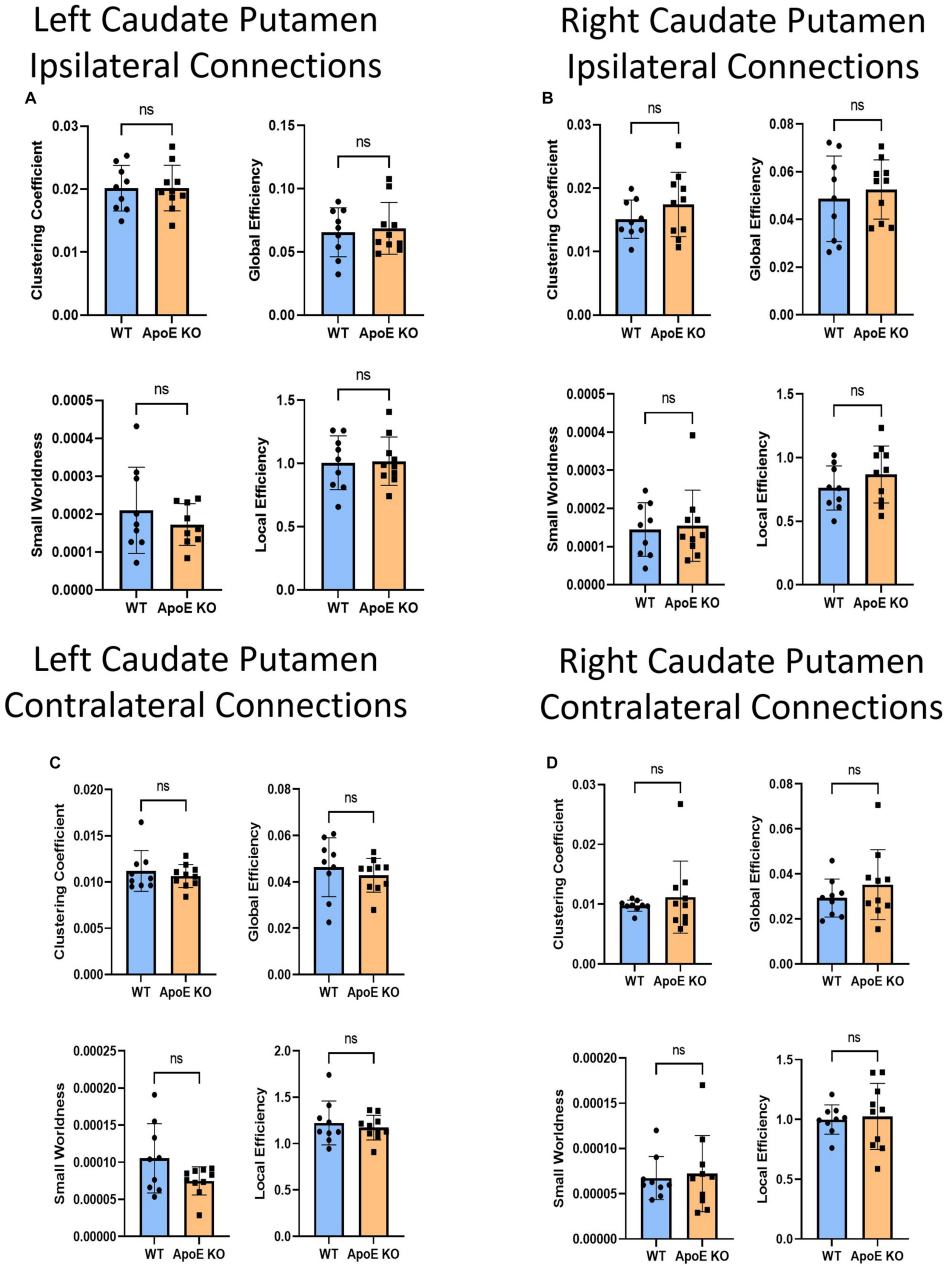
Average network parameters derived from caudate putamen-seeded fiber tracking for left ipsilateral (**A**, top right: clustering coefficient, bottom right: small worldness, top left: global efficiency and bottom left: local efficiency), right ipsilateral **(B)**, left contralateral **(C),** and right contralateral **(D)** are compared for WT and ApoE KO, *t*-test was used to determine significance.

Our whole hemisphere unbiased approach revealed two areas of significant differences between the CP and other regions in the left hemisphere, and 3 in the right hemisphere. While these are fewer differences in connectivity than the hippocampus and amygdala, we conducted ipsilateral fiber tracking placing seeds in the left or right CP and mapping to the rest of the respective hemisphere to see if differences in the ROI fibers persisted. There was no significant difference in average fiber numbers (Table 4). See Supplementary Figure S5 for more details. While there was no difference in overall connectivity between ApoE KO and WT CP fibers, we did find one region with significantly lower numbers of fiber connections in ApoE KO than WT (Table 5). When comparing ApoE KO and WT network parameters we found no significant differences in clustering coefficient (WT: 0.020 ± 0.0036, ApoE KO: 0.020 ± 0.0036, *p* = 0.98), small worldness (WT: 0.00021 ± 0.00011, ApoE KO: 0.00021 ± 0.00013, *p* = 0.99), local efficiency (WT: 1.00 ± 0.21, ApoE KO: 1.02 ± 0.19, *p* = 0.90), or global efficiency (WT: 0.066 ± 0.019, ApoE KO: 0.069 ± 0.021, *p* = 0.74) (Figure 7A). When comparing ApoE KO and WT network parameters we found no significant differences in clustering coefficient (WT: 0.020 ± 0.0036, ApoE KO: 0.020 ± 0.0036, *p* = 0.98), small worldness (WT: 0.00021 ± 0.00011, ApoE KO: 0.00021 ± 0.00013, *p* = 0.99), local efficiency (WT: 1.00 ± 0.21, ApoE KO: 1.02 ± 0.19, *p* = 0.90), or global efficiency (WT: 0.066 ± 0.019, ApoE KO: 0.069 ± 0.021, *p* = 0.74).

When placing fiber seeds in the right CP, we again found no significant difference in average fiber number (Table 4), however we found that WT had significantly higher fiber numbers in 5 connecting regions (Table 5). Interestingly, these differences in connectivity all involved the amygdala. When comparing ApoE KO and WT network parameters we found no significant differences in clustering coefficient (WT: 0.015 ± 0.0030, ApoE KO: 0.017 ± 0.0051, *p* = 0.25), small worldness (WT: 0.00014 ± 7.02E-05, ApoE KO: 0.00015 ± 9.33E-05, *p* = 0.80), local efficiency (WT: 0.76 ± 0.17, ApoE KO: 0.87 ± 0.22, *p* = 0.26), or global efficiency (WT: 0.049 ± 0.018, ApoE KO: 0.053 ± 0.012, *p* = 0.58) (Figure 7B).

#### 3.1.8 ApoE KO vs. WT contralateral caudate putamen analysis

To explore ApoE’s effects on long-range neuronal development in the caudate putamen (CP), we planted seeds in the left CP and mapped both local connections to other regions in the left hemisphere, and mapped their connections to the right hemisphere, for a total of 72 possible connections (see Supplementary Figure S6). There was no significant difference in average fiber number between ApoE KO and WT and there were no significant differences between connecting regions (Table 4). We compared ApoE KO and WT network parameters and found no significant differences between 4 different parameters (Figure 7C) clustering coefficient (WT: 0.0011 ± 0.0022, ApoE KO: 0.011 ± 0.0012, *p* = 0.51), Small worldness (WT: 0.0001053 ± 4.672E-05, ApoE KO: 7.485E-05 ± 1.897E-05, *p* = 0.075), local efficiency (WT: 1.22 ± 0.24, ApoE KO: 1.17 ± 0.13, *p* = 0.57), and global efficiency (WT: 0.046 ± 0.013, ApoE KO: 0.043 ± 0.0072, *p* = 0.47).

We explored long-range connectivity in the caudate putamen on the right side as well (RCP). There were no significant differences between average fiber number or between connecting regions (Table 4). We compared ApoE KO and WT network parameters and found that clustering coefficient (WT: 0.0098 ± 0.00094, ApoE KO: 0.011 ± 0.0060, *p* = 0.49), small worldness (WT: 0.6.7E-05 ± 2.37E-05, ApoE KO: 7.23E-05 ± 4.22E-05, *p* = 0.76), local efficiency (WT: 1.00 ± 0.12, ApoE KO: 1.03 ± 0.28, *p* = 0.79), and global efficiency (WT: 0.029 ± 0.0084, ApoE KO: 0.035 ± 0.016, *p* = 0.32) were not significantly different (Figure 7D).

## 4 Discussion

Our study found that ApoE deficiency in five-month-old adult mice resulted in region-specific brain network remodeling, not in the hemisphere, but specifically in the fiber tracts connecting the hippocampus and amygdala regions to the other ipsilateral regions. These brain regions are associated with learning and memory and anxiety. The fiber remodeling in the caudate putamen looked different than that of the hippocampus and amygdala, with fewer significant differences and no changes in network parameters. Each hemisphere as a single ROI is unlikely to lead to globally detectable differences. Though ApoE is found in many areas of the brain, it is localized in the olfactory bulb, nerves and pathways as well as in the hippocampus and amygdala, specifically (Boyles et al., 1985; Grehan et al., 2001). ApoE KO mice are not known to exhibit any locomotor or appetitive deficits (Oitzl et al., 1997; Raber et al., 2000), and behaved similarly to wildtype in sleep and coordination tests (Fuentes et al., 2018), so it would follow that brain processing in most ROIs, when the hemisphere is treated as one, should not look entirely different.

When placing fiber tracking seeds in the hippocampus and amygdala specifically, however, we saw evidence of brain fiber network remodeling. We intentionally used mice of mature adult age, such they would not exhibit age-related phenotypes yet, allowing us to study the effects of ApoE on network topology without compounding the effects of aging. Seven and ten connecting regions had significantly lower numbers of connections in ApoE KO than WT in the hippocampus and amygdala, respectively. This in combination with decreased clustering coefficient, small worldness, global efficiency and local efficiency, indicates fiber network deficiencies in the ApoE KO mice. To some extent, we would expect to see differences in brain fiber network, especially in brain regions related to olfactory processing, working memory, and exploratory behaviors. Nathan et al. (2004) found that APOE KO mice have deficient olfactory function and could not differentiate between water and odorant solution even when an aversive taste was added (Nathan et al., 2004). Gordon et al. (1995) found that during the Morris Water Maze, ApoE KO mice’s latency to the platform between trials one and two on a given day did not improve, suggesting impaired working memory process (Gordon et al., 1995). Further evidence for ApoE KO decreased working memory and exploratory behavior came from Fuentes et al. (2018), who found that ApoE KO spent less time exploring the novel arm during the forced alternation test, and also that ApoE KO had a lower preference for the novel object in the novel object recognition test (Fuentes et al., 2018). The olfactory pathway related to averse or predator scents involves the amygdala (de Castro, 2009), and spatial and working memory are linked to the hippocampus, individual pathway analysis should reveal differences in these areas (D'Hooge and De Deyn, 2001).

The quality of these connections and the way in which they affect the functional integration and efficiency of the network differed between ApoE KO and WT for both ROI pathways we looked at. Small worldness and global efficiency are measures of network efficiency (Rubinov and Sporns, 2010; Scharwächter et al., 2022), both of which were significantly lower in the hippocampus- and amygdala-seeded pathways of ApoE KO mice. Measures of network efficiency provide insight into how well subunits of a network are integrated, allowing for information processing (Rubinov and Sporns, 2010; Scharwächter et al., 2022). Small worldness informs of how nodes are connected locally as opposed to how a random network is connected. A tell-tale sign of this interconnectedness is high clustering with a minimum path length, as it predicts more specifically designed information sharing between neighbors, especially if this distribution occurs across the entire network (Watts and Strogatz, 1998; Achard et al., 2006). Global efficiency was significantly lower for the hippocampus and amygdala fiber tract pathways and quantifies how regions communicate with each other (Rubinov and Sporns, 2010; Ingalhalikar et al., 2015). In humans, a decrease in global efficiency is associated with dementia and cognitive impairment in certain patients (Lawrence et al., 2014; Berlot et al., 2016), and we see it affected in regions where ApoE is known to localize.

High functioning networks require integration and efficiency, but likewise tend to divide into subunits, known as network segregation (Rubinov and Sporns, 2010). Random networks will have low clustering coefficients, since they are the number of actual connections between neighboring nodes versus the number of possible connections. High clustering coefficient in tandem with high local efficiency indicates that information transfer between nearby nodes is efficient and effective (Basser and Pierpaoli, 1996; Watts and Strogatz, 1998; Rubinov and Sporns, 2010; Ingalhalikar et al., 2015). Clustering coefficient and local efficiency are significantly lower in ApoE KO hippocampus- and amygdala-seeded pathways and indicate that individual subunits of their network are not structured to communicate as efficiently as their WT counterparts.

When it comes to differences in the caudate putamen-seeded fiber tracts, we did not see differences in network parameters or overall fiber numbers, but we did some differences in specific connection regions. Interestingly, all of these differences involved the amygdala. The caudate putamen plays a central role in integrating information particularly pertaining to cognition and emotion, performing learned movements, and is also linked to dopaminergic reward pathways (Schröder et al., 2020). While there are some inconsistent findings, generally decreased levels of dopaminergic neurotransmitters have been linked to Alzheimer’s disorder (Pan et al., 2019). While our analysis cannot pinpoint specific types of neurotransmitters, decreases in connectivity specific to the amygdala is an interesting finding. It could suggest that neuronal development involving these integral brain regions is altered by a loss of ApoE. ApoE KO mice are a widely studied animal model, both as AD models as well as atherosclerosis models. Other mouse models of AD exist, and have been studied using diffusion MRI, and while the results were inconsistent in some regards, a common finding was that DTI identified altered diffusion in gray matter (Jullienne et al., 2022). Better characterization of mouse models of disease only makes it more possible to translate findings into human data. Our study is limited to *ex-vivo* male mice, therefore does not encapsulate these characterizations in female ApoE KO neuronal network patterning or *in-vivo* functional data. However, by focusing in on the network organization of one hemisphere at a time, we were able to determine the differences in connections.

Our study found that ApoE affects network patterning. This is consistent with the observations of altered network patterning in old mice carrying human ApoE isoforms (Badea et al., 2019). However, in the study of old mice, it is unclear if ApoE is affecting neurodevelopment of network or the degeneration of the network. In our study, the five-month-of-age in mice is equivalent to mature adults in humans, an age that is much earlier than the equivalent age of LOAD onset. This suggests that LOAD vulnerability or resiliency could have already existed long before manifestation of LOAD symptoms, suggesting a neurodevelopment origin of LOAD vulnerability.

Although ApoE polymorphism is known to increase or decrease LOAD risks in an allele-specific manner, carrying either of the three genetic variants cannot predict if an individual will or will not develop LOAD later in life. Our study proves useful in finding changes in graph theory related to disease phenotype, which is already done in human studies (Lawrence et al., 2014; Berlot et al., 2016). The diffusion parameters and brain network characteristics derived in our mouse study can be non-invasively and quantitatively obtained in humans. Our study suggests that brain network characteristics might be possible surrogate biomarkers in conjunction of ApoE polymorphism in prediction of LOAD in each individual. If the brain network characteristics of individuals carrying ε2, ε3, or ε4 isoforms with or without LOAD can be established at a young disease-free stage and an old diseased age, these can potentially offer a pathway for future individualized precision medicine for potential early detection and intervention in the sub-clinical phase before downstream pathological features of amyloid plaques or neurofibrillary tangles occur.

## 5 Conclusion

We used diffusion tensor MRI in conjunction of graph theory to show that a deficiency of Apolipoprotien-E (ApoE) leads to altered brain network, especially in the network reflected in the hippocampus, amygdala, and caudate putamen, in five-month-old adult mice. Network topology based on graph theory of ApoE KO single hemisphere analysis demonstrated decreased functional integration, network efficiency, and network segregation between the hippocampus and amygdala and the rest of the brain, with significantly lower local and global efficiency, small worldness and clustering coefficient, as compared to WT, depending on hemisphere. Whole-brain seeding for fiber tracts did not reveal any significant changes between WT and ApoE KO, however, when looking individually into brain regions and pathway known to be affected by LOAD and where ApoE is known to localize, network topology is significantly different. Our findings suggest a possible neurodevelopmental origin of vulnerability by ApoE deficiency through altered brain network structures, long before the equivalent age of LOAD onset. Our data showed that brain network developed differently in ApoE KO and WT mice, indicating that ApoE is involved in brain network development. This suggests a possibility that ApoE might attenuate risks of LOAD *via* altering brain network architecture. The three-month-of-age in mice is equivalent to young adults in humans, an age that is much earlier than the equivalent age of LOAD onset. This suggests that LOAD vulnerability or resiliency could have already existed long before manifestation of LOAD symptoms.

## Data availability statement

The raw data supporting the conclusions of this article will be made available by the authors, without undue reservation.

## Ethics statement

The animal study was approved by University of Pittsburgh Institutional Animal Care and Use Committee. The study was conducted in accordance with the local legislation and institutional requirements.

## Author contributions

MS and SW harvested brain samples. MS bred the animals for the study, performed the neuronal network analysis, and wrote the manuscript. SW and KS performed the image acquisition. SK, DC, SM, and PB-S performed the registration to Allen atlas. CM performed the statistical analysis. YW was responsible for overall study design and overseeing all aspects of the study. All authors contributed to the article and approved the submitted version.

## Footnotes

^1^ https://dsi-studio.labsolver.org/

^2^ http://circos.ca/
