# Supplementary material for: Apolipoprotein-E deficiency leads to brain network alteration characterized by diffusion MRI and graph theory
Source: Front Neurosci. 2023 Nov 21;17:1183312. doi: 10.3389/fnins.2023.1183312 (PMC10702609; doi:10.3389/fnins.2023.1183312)
Supplement: Supplementary file 1 [file Table_1.docx]

Supplementary Table 1. Wildtype diffusion parameters for each region in the left and right hemisphere for the atlas based parcellation are listed with standard deviation and FDR adjusted p-values after t-test.

| FA | | | | | AD | | | | | MD | | | | | RD | | | | | Volume | | | | |  |
| --- | --- | --- | --- | --- | --- | --- | --- | --- | --- | --- | --- | --- | --- | --- | --- | --- | --- | --- | --- | --- | --- | --- | --- | --- | --- |
| FDR Adjusted P value | SD | Right Hemisphere Mean | SD | Left Hemisphere Mean | FDR Adjusted P value | SD | Right Hemisphere Mean | SD | Left Hemisphere Mean | FDR Adjusted P value | SD | Right Hemisphere Mean | SD | Left Hemisphere Mean | FDR Adjusted P value | SD | Right Hemisphere Mean | SD | Left Hemisphere Mean | FDR Adjusted P value | SD | Right Hemisphere Mean | SD | Left Hemisphere Mean |  |
| 0.9905 | 0.10 | 0.46 | 0.10 | 0.46 | 0.9415 | 0.03 | 0.34 | 0.04 | 0.35 | >0.999999 | 0.04 | 0.23 | 0.04 | 0.23 | >0.999999 | 0.04 | 0.18 | 0.04 | 0.18 | 0.846 | 0.32 | 3.48 | 0.26 | 3.41 | CC |
| 0.9826 | 0.11 | 0.28 | 0.07 | 0.23 | 0.8737 | 0.05 | 0.33 | 0.03 | 0.34 | 0.9395 | 0.05 | 0.26 | 0.03 | 0.28 | 0.9479 | 0.06 | 0.23 | 0.03 | 0.25 | 0.846 | 0.64 | 12.04 | 0.42 | 12.17 | CP |
| 0.9826 | 0.11 | 0.52 | 0.06 | 0.47 | 0.8355 | 0.03 | 0.37 | 0.04 | 0.40 | 0.7893 | 0.04 | 0.23 | 0.03 | 0.25 | 0.8524 | 0.04 | 0.16 | 0.03 | 0.18 | 0.9248 | 0.04 | 0.37 | 0.04 | 0.36 | ACOL |
| 0.9826 | 0.08 | 0.36 | 0.04 | 0.31 | 0.8355 | 0.03 | 0.33 | 0.03 | 0.35 | 0.7893 | 0.04 | 0.24 | 0.02 | 0.26 | 0.8524 | 0.04 | 0.20 | 0.02 | 0.22 | 0.9376 | 0.23 | 4.35 | 0.25 | 4.37 | PAL |
| 0.9826 | 0.12 | 0.65 | 0.08 | 0.63 | 0.5908 | 0.07 | 0.40 | 0.04 | 0.45 | 0.7358 | 0.05 | 0.22 | 0.03 | 0.25 | 0.8524 | 0.05 | 0.13 | 0.03 | 0.15 | >0.999999 | 0.08 | 0.82 | 0.07 | 0.82 | IntC |
| 0.9826 | 0.14 | 0.37 | 0.11 | 0.35 | 0.8355 | 0.05 | 0.33 | 0.03 | 0.35 | 0.9422 | 0.06 | 0.24 | 0.04 | 0.26 | >0.999999 | 0.06 | 0.20 | 0.05 | 0.21 | 0.7473 | 0.35 | 9.86 | 0.28 | 9.74 | TH |
| 0.9826 | 0.05 | 0.27 | 0.06 | 0.28 | 0.8737 | 0.01 | 0.39 | 0.03 | 0.38 | 0.9422 | 0.02 | 0.30 | 0.03 | 0.30 | >0.999999 | 0.02 | 0.26 | 0.04 | 0.26 | 0.9146 | 0.80 | 23.83 | 0.75 | 23.71 | CB |
| 0.9826 | 0.14 | 0.30 | 0.11 | 0.28 | 0.8737 | 0.04 | 0.30 | 0.04 | 0.31 | 0.9422 | 0.05 | 0.24 | 0.04 | 0.24 | >0.999999 | 0.05 | 0.20 | 0.05 | 0.21 | 0.846 | 0.29 | 3.27 | 0.20 | 3.22 | SUC |
| 0.9826 | 0.09 | 0.20 | 0.08 | 0.21 | 0.118 | 0.11 | 0.89 | 0.11 | 0.71 | 0.4689 | 0.12 | 0.76 | 0.11 | 0.61 | 0.8524 | 0.13 | 0.70 | 0.12 | 0.56 | 0.6128 | 0.25 | 2.86 | 0.23 | 3.01 | VS |
| 0.9826 | 0.06 | 0.27 | 0.05 | 0.26 | 0.8304 | 0.02 | 0.31 | 0.03 | 0.33 | 0.7358 | 0.02 | 0.25 | 0.02 | 0.26 | 0.8524 | 0.02 | 0.21 | 0.02 | 0.23 | 0.6128 | 0.42 | 7.53 | 0.40 | 7.30 | HY |
| 0.9826 | 0.10 | 0.27 | 0.08 | 0.26 | 0.8737 | 0.03 | 0.35 | 0.04 | 0.34 | 0.9422 | 0.04 | 0.28 | 0.05 | 0.27 | >0.999999 | 0.04 | 0.25 | 0.05 | 0.24 | 0.7473 | 0.15 | 1.94 | 0.11 | 1.88 | IC |
| 0.9826 | 0.17 | 0.30 | 0.14 | 0.28 | 0.8737 | 0.05 | 0.29 | 0.04 | 0.29 | >0.999999 | 0.04 | 0.23 | 0.05 | 0.23 | >0.999999 | 0.05 | 0.20 | 0.05 | 0.20 | 0.6404 | 0.10 | 2.03 | 0.11 | 1.97 | PAG |
| 0.9826 | 0.08 | 0.25 | 0.08 | 0.24 | 0.8737 | 0.02 | 0.34 | 0.03 | 0.34 | 0.9738 | 0.03 | 0.28 | 0.03 | 0.28 | >0.999999 | 0.03 | 0.25 | 0.04 | 0.25 | 0.6128 | 1.25 | 28.14 | 1.03 | 26.99 | ICTx |
| 0.9826 | 0.07 | 0.23 | 0.06 | 0.19 | 0.8737 | 0.04 | 0.38 | 0.06 | 0.39 | 0.883 | 0.04 | 0.31 | 0.04 | 0.33 | 0.8524 | 0.04 | 0.28 | 0.04 | 0.30 | 0.6128 | 0.21 | 2.68 | 0.18 | 2.57 | COA |
| 0.9826 | 0.05 | 0.25 | 0.05 | 0.25 | 0.8737 | 0.02 | 0.41 | 0.02 | 0.41 | >0.999999 | 0.02 | 0.32 | 0.02 | 0.32 | >0.999999 | 0.02 | 0.28 | 0.02 | 0.28 | 0.7022 | 0.97 | 11.62 | 1.58 | 11.02 | OlfA |
| 0.9826 | 0.08 | 0.31 | 0.05 | 0.30 | 0.4147 | 0.02 | 0.35 | 0.03 | 0.38 | 0.5963 | 0.02 | 0.27 | 0.01 | 0.29 | 0.8524 | 0.03 | 0.22 | 0.01 | 0.24 | 0.7473 | 0.40 | 7.60 | 0.20 | 7.73 | P |
| 0.9826 | 0.17 | 0.36 | 0.10 | 0.33 | 0.8737 | 0.05 | 0.31 | 0.03 | 0.32 | 0.9422 | 0.05 | 0.23 | 0.03 | 0.24 | >0.999999 | 0.06 | 0.19 | 0.04 | 0.20 | 0.6128 | 0.15 | 2.12 | 0.11 | 2.03 | RA |
| 0.9826 | 0.06 | 0.24 | 0.02 | 0.19 | 0.8355 | 0.04 | 0.36 | 0.03 | 0.38 | 0.7358 | 0.04 | 0.29 | 0.03 | 0.32 | 0.8524 | 0.04 | 0.26 | 0.02 | 0.29 | 0.846 | 0.15 | 2.16 | 0.16 | 2.20 | NA |
| >0.999999 | 0.15 | 0.67 | 0.11 | 0.67 | 0.8737 | 0.17 | 0.42 | 0.13 | 0.40 | 0.9422 | 0.13 | 0.26 | 0.10 | 0.22 | >0.999999 | 0.12 | 0.17 | 0.09 | 0.14 | 0.6128 | 0.08 | 0.73 | 0.06 | 0.66 | F |
| 0.9826 | 0.10 | 0.27 | 0.08 | 0.25 | 0.8355 | 0.03 | 0.31 | 0.04 | 0.33 | 0.9395 | 0.04 | 0.25 | 0.04 | 0.27 | >0.999999 | 0.04 | 0.22 | 0.04 | 0.24 | 0.6128 | 0.21 | 2.55 | 0.13 | 2.67 | ACA |
| 0.9826 | 0.05 | 0.20 | 0.06 | 0.21 | 0.8355 | 0.02 | 0.32 | 0.03 | 0.33 | 0.9422 | 0.02 | 0.27 | 0.03 | 0.28 | >0.999999 | 0.02 | 0.24 | 0.04 | 0.25 | 0.6128 | 0.95 | 11.22 | 1.09 | 11.82 | MO |
| 0.9826 | 0.07 | 0.23 | 0.12 | 0.24 | 0.8737 | 0.02 | 0.32 | 0.04 | 0.33 | 0.9422 | 0.02 | 0.27 | 0.04 | 0.27 | >0.999999 | 0.03 | 0.24 | 0.05 | 0.24 | 0.6128 | 0.84 | 14.99 | 0.84 | 14.49 | SS |
| 0.9826 | 0.07 | 0.24 | 0.05 | 0.20 | 0.8355 | 0.05 | 0.39 | 0.05 | 0.41 | 0.7893 | 0.04 | 0.31 | 0.03 | 0.34 | 0.8524 | 0.04 | 0.27 | 0.03 | 0.30 | 0.7473 | 0.34 | 5.33 | 0.30 | 5.21 | PIR |
| 0.9826 | 0.06 | 0.25 | 0.03 | 0.22 | 0.8355 | 0.04 | 0.35 | 0.04 | 0.37 | 0.883 | 0.04 | 0.28 | 0.03 | 0.30 | 0.8524 | 0.04 | 0.25 | 0.03 | 0.27 | 0.6128 | 0.23 | 3.31 | 0.27 | 3.13 | TT |
| 0.9826 | 0.07 | 0.21 | 0.14 | 0.25 | 0.8737 | 0.06 | 0.37 | 0.04 | 0.36 | 0.883 | 0.04 | 0.31 | 0.04 | 0.28 | 0.8524 | 0.04 | 0.28 | 0.05 | 0.25 | 0.8615 | 0.01 | 0.07 | 0.01 | 0.08 | MOB_gl |
| 0.9826 | 0.05 | 0.28 | 0.07 | 0.27 | 0.8355 | 0.03 | 0.38 | 0.05 | 0.36 | 0.883 | 0.03 | 0.29 | 0.03 | 0.28 | 0.9479 | 0.03 | 0.25 | 0.03 | 0.24 | 0.7022 | 0.02 | 0.11 | 0.02 | 0.10 | MOB_gr |
| 0.9826 | 0.09 | 0.29 | 0.07 | 0.25 | 0.8737 | 0.03 | 0.35 | 0.03 | 0.34 | >0.999999 | 0.04 | 0.27 | 0.04 | 0.27 | >0.999999 | 0.04 | 0.24 | 0.04 | 0.24 | 0.7022 | 0.27 | 3.46 | 0.26 | 3.34 | RHP |
| 0.9826 | 0.09 | 0.26 | 0.08 | 0.25 | 0.8737 | 0.03 | 0.35 | 0.03 | 0.36 | 0.9422 | 0.03 | 0.28 | 0.03 | 0.29 | >0.999999 | 0.04 | 0.24 | 0.04 | 0.25 | 0.6128 | 0.75 | 5.38 | 0.17 | 4.92 | EC |
| 0.9826 | 0.08 | 0.28 | 0.10 | 0.29 | 0.8355 | 0.03 | 0.37 | 0.03 | 0.39 | 0.9422 | 0.04 | 0.29 | 0.04 | 0.30 | >0.999999 | 0.04 | 0.25 | 0.05 | 0.25 | 0.6128 | 0.40 | 4.93 | 0.29 | 4.62 | CA1 |
| 0.9826 | 0.10 | 0.31 | 0.09 | 0.28 | 0.9365 | 0.03 | 0.39 | 0.03 | 0.40 | 0.9422 | 0.04 | 0.30 | 0.03 | 0.31 | >0.999999 | 0.05 | 0.26 | 0.04 | 0.27 | 0.6128 | 0.21 | 3.06 | 0.16 | 2.87 | CA3 |
| 0.9826 | 0.09 | 0.28 | 0.10 | 0.29 | 0.8737 | 0.03 | 0.40 | 0.03 | 0.40 | >0.999999 | 0.04 | 0.31 | 0.04 | 0.31 | >0.999999 | 0.05 | 0.27 | 0.05 | 0.27 | 0.6128 | 0.19 | 3.38 | 0.14 | 3.28 | DG |
| 0.9826 | 0.11 | 0.31 | 0.12 | 0.33 | >0.999999 | 0.03 | 0.38 | 0.03 | 0.38 | 0.9738 | 0.04 | 0.29 | 0.04 | 0.29 | >0.999999 | 0.05 | 0.25 | 0.05 | 0.24 | 0.846 | 0.04 | 0.30 | 0.02 | 0.31 | CA2 |
| 0.9826 | 0.06 | 0.25 | 0.05 | 0.23 | 0.8355 | 0.04 | 0.38 | 0.04 | 0.36 | 0.9422 | 0.03 | 0.31 | 0.04 | 0.30 | >0.999999 | 0.03 | 0.27 | 0.03 | 0.26 | 0.9248 | 0.02 | 0.13 | 0.02 | 0.13 | MOB_mi |
| 0.9826 | 0.08 | 0.28 | 0.06 | 0.25 | 0.8355 | 0.04 | 0.34 | 0.03 | 0.36 | 0.8102 | 0.04 | 0.27 | 0.02 | 0.29 | 0.8524 | 0.04 | 0.23 | 0.03 | 0.26 | 0.846 | 0.38 | 6.95 | 0.45 | 6.84 | STR |
| 0.9826 | 0.13 | 0.37 | 0.08 | 0.34 | 0.8355 | 0.03 | 0.32 | 0.02 | 0.33 | 0.883 | 0.04 | 0.23 | 0.03 | 0.25 | 0.9631 | 0.05 | 0.19 | 0.03 | 0.21 | 0.6128 | 0.35 | 6.64 | 0.26 | 6.43 | MB |
| 0.9826 | 0.06 | 0.28 | 0.03 | 0.26 | 0.8737 | 0.02 | 0.38 | 0.03 | 0.39 | 0.9422 | 0.02 | 0.29 | 0.02 | 0.30 | 0.9631 | 0.03 | 0.25 | 0.02 | 0.26 | 0.9248 | 2.40 | 14.40 | 1.65 | 14.61 | MY |
