# Supplementary material for: Apolipoprotein-E deficiency leads to brain network alteration characterized by diffusion MRI and graph theory
Source: Front Neurosci. 2023 Nov 21;17:1183312. doi: 10.3389/fnins.2023.1183312 (PMC10702609; doi:10.3389/fnins.2023.1183312)
Supplement: Supplementary file 2 [file Table_2.docx]

Supplementary Table 2. ApoE KO diffusion parameters for each region in the left and right hemisphere for the atlas based parcellation are listed with standard deviation and FDR adjusted p-values after t-test.

| FA | | | | | AD | | | | | MD | | | | | | RD | | | | | | Volume | | | | | |  |
| --- | --- | --- | --- | --- | --- | --- | --- | --- | --- | --- | --- | --- | --- | --- | --- | --- | --- | --- | --- | --- | --- | --- | --- | --- | --- | --- | --- | --- |
| FDR Adjusted P value | SD | Right Hemisphere Mean | SD | Left Hemisphere Mean | FDR Adjusted P value | SD | Right Hemisphere Mean | SD | Left Hemisphere Mean | FDR Adjusted P value | SD | Right Hemisphere Mean | SD | Left Hemisphere Mean | FDR Adjusted P value | | SD | Right Hemisphere Mean | SD | Left Hemisphere Mean | FDR Adjusted P value | | SD | Right Hemisphere Mean | SD | Left Hemisphere Mean |  | |
| 0.6616 | 0.02 | 0.37 | 0.03 | 0.39 | >0.999999 | 0.06 | 0.38 | 0.04 | 0.39 | >0.999999 | 0.05 | 0.27 | 0.04 | 0.28 | >0.999999 | | 0.05 | 0.22 | 0.03 | 0.22 | >0.999999 | | 0.48 | 3.40 | 0.45 | 3.32 | CC | |
| 0.9158 | 0.03 | 0.19 | 0.04 | 0.19 | >0.999999 | 0.04 | 0.34 | 0.02 | 0.33 | >0.999999 | 0.03 | 0.28 | 0.02 | 0.27 | >0.999999 | | 0.03 | 0.26 | 0.02 | 0.25 | >0.999999 | | 1.08 | 12.01 | 1.17 | 11.96 | CP | |
| >0.999999 | 0.06 | 0.45 | 0.06 | 0.45 | >0.999999 | 0.02 | 0.37 | 0.03 | 0.37 | >0.999999 | 0.01 | 0.24 | 0.01 | 0.24 | >0.999999 | | 0.02 | 0.17 | 0.02 | 0.17 | >0.999999 | | 0.03 | 0.34 | 0.03 | 0.34 | ACOL | |
| 0.6616 | 0.02 | 0.30 | 0.02 | 0.29 | >0.999999 | 0.02 | 0.34 | 0.01 | 0.34 | >0.999999 | 0.02 | 0.26 | 0.00 | 0.26 | >0.999999 | | 0.02 | 0.22 | 0.00 | 0.22 | >0.999999 | | 0.22 | 4.34 | 0.21 | 4.34 | PAL | |
| 0.7047 | 0.05 | 0.59 | 0.05 | 0.61 | >0.999999 | 0.03 | 0.45 | 0.03 | 0.46 | >0.999999 | 0.01 | 0.25 | 0.01 | 0.26 | >0.999999 | | 0.02 | 0.16 | 0.01 | 0.16 | >0.999999 | | 0.07 | 0.87 | 0.08 | 0.90 | IntC | |
| 0.6616 | 0.02 | 0.26 | 0.02 | 0.27 | >0.999999 | 0.01 | 0.34 | 0.02 | 0.35 | >0.999999 | 0.01 | 0.27 | 0.02 | 0.27 | >0.999999 | | 0.01 | 0.23 | 0.01 | 0.23 | >0.999999 | | 0.51 | 9.79 | 0.56 | 9.62 | TH | |
| 0.9515 | 0.02 | 0.22 | 0.03 | 0.23 | >0.999999 | 0.02 | 0.38 | 0.01 | 0.37 | >0.999999 | 0.02 | 0.31 | 0.01 | 0.30 | >0.999999 | | 0.02 | 0.27 | 0.01 | 0.27 | >0.999999 | | 1.36 | 23.16 | 0.89 | 22.91 | CB | |
| 0.9515 | 0.01 | 0.20 | 0.02 | 0.20 | >0.999999 | 0.01 | 0.32 | 0.02 | 0.33 | >0.999999 | 0.01 | 0.27 | 0.01 | 0.27 | >0.999999 | | 0.01 | 0.24 | 0.01 | 0.25 | >0.999999 | | 0.28 | 3.24 | 0.13 | 3.22 | SUC | |
| 0.6616 | 0.01 | 0.14 | 0.01 | 0.15 | >0.999999 | 0.23 | 0.86 | 0.20 | 0.78 | >0.999999 | 0.20 | 0.77 | 0.17 | 0.69 | >0.999999 | | 0.19 | 0.72 | 0.16 | 0.65 | >0.999999 | | 0.47 | 2.81 | 0.53 | 3.07 | VS | |
| 0.6616 | 0.02 | 0.22 | 0.01 | 0.23 | >0.999999 | 0.02 | 0.31 | 0.02 | 0.32 | >0.999999 | 0.02 | 0.26 | 0.01 | 0.26 | >0.999999 | | 0.01 | 0.23 | 0.01 | 0.23 | >0.999999 | | 0.39 | 7.46 | 0.31 | 7.30 | HY | |
| 0.8837 | 0.02 | 0.22 | 0.03 | 0.21 | >0.999999 | 0.02 | 0.35 | 0.02 | 0.35 | >0.999999 | 0.02 | 0.29 | 0.01 | 0.29 | >0.999999 | | 0.02 | 0.26 | 0.01 | 0.26 | >0.999999 | | 0.12 | 1.87 | 0.10 | 1.89 | IC | |
| 0.6616 | 0.02 | 0.19 | 0.01 | 0.18 | >0.999999 | 0.01 | 0.30 | 0.01 | 0.30 | >0.999999 | 0.01 | 0.25 | 0.01 | 0.25 | >0.999999 | | 0.01 | 0.23 | 0.01 | 0.23 | >0.999999 | | 0.13 | 1.98 | 0.14 | 1.96 | PAG | |
| 0.7924 | 0.02 | 0.17 | 0.03 | 0.18 | >0.999999 | 0.02 | 0.34 | 0.03 | 0.36 | >0.999999 | 0.02 | 0.29 | 0.03 | 0.30 | >0.999999 | | 0.02 | 0.27 | 0.03 | 0.28 | >0.999999 | | 1.16 | 26.63 | 1.28 | 26.65 | ICTx | |
| >0.999999 | 0.04 | 0.17 | 0.04 | 0.17 | >0.999999 | 0.03 | 0.37 | 0.03 | 0.36 | >0.999999 | 0.02 | 0.32 | 0.02 | 0.31 | >0.999999 | | 0.02 | 0.29 | 0.02 | 0.28 | >0.999999 | | 0.36 | 2.77 | 0.13 | 2.62 | COA | |
| 0.7924 | 0.02 | 0.20 | 0.02 | 0.21 | >0.999999 | 0.02 | 0.39 | 0.02 | 0.39 | >0.999999 | 0.02 | 0.32 | 0.02 | 0.32 | >0.999999 | | 0.02 | 0.29 | 0.02 | 0.29 | >0.999999 | | 1.31 | 10.12 | 0.87 | 10.33 | OlfA | |
| 0.6616 | 0.02 | 0.26 | 0.03 | 0.28 | >0.999999 | 0.02 | 0.36 | 0.01 | 0.36 | >0.999999 | 0.01 | 0.28 | 0.01 | 0.28 | >0.999999 | | 0.01 | 0.24 | 0.01 | 0.24 | >0.999999 | | 0.45 | 7.42 | 0.40 | 7.31 | P | |
| 0.6616 | 0.02 | 0.26 | 0.02 | 0.25 | >0.999999 | 0.01 | 0.33 | 0.01 | 0.33 | >0.999999 | 0.01 | 0.25 | 0.01 | 0.26 | >0.999999 | | 0.01 | 0.22 | 0.01 | 0.22 | >0.999999 | | 0.14 | 2.05 | 0.12 | 2.11 | RA | |
| 0.9515 | 0.02 | 0.18 | 0.03 | 0.18 | >0.999999 | 0.02 | 0.33 | 0.02 | 0.33 | >0.999999 | 0.02 | 0.28 | 0.02 | 0.28 | >0.999999 | | 0.02 | 0.26 | 0.02 | 0.25 | >0.999999 | | 0.12 | 2.10 | 0.09 | 2.11 | NA | |
| 0.9515 | 0.11 | 0.61 | 0.13 | 0.62 | >0.999999 | 0.20 | 0.54 | 0.23 | 0.55 | >0.999999 | 0.20 | 0.32 | 0.23 | 0.33 | >0.999999 | | 0.20 | 0.22 | 0.24 | 0.22 | >0.999999 | | 0.17 | 0.75 | 0.16 | 0.70 | F | |
| 0.819 | 0.02 | 0.19 | 0.03 | 0.20 | >0.999999 | 0.02 | 0.31 | 0.02 | 0.32 | >0.999999 | 0.01 | 0.26 | 0.02 | 0.26 | >0.999999 | | 0.01 | 0.24 | 0.02 | 0.24 | >0.999999 | | 0.20 | 2.57 | 0.25 | 2.54 | ACA | |
| 0.6689 | 0.01 | 0.16 | 0.03 | 0.17 | >0.999999 | 0.01 | 0.32 | 0.01 | 0.32 | >0.999999 | 0.01 | 0.28 | 0.01 | 0.28 | >0.999999 | | 0.01 | 0.25 | 0.02 | 0.25 | >0.999999 | | 0.84 | 11.30 | 0.76 | 11.23 | MO | |
| 0.6616 | 0.02 | 0.15 | 0.04 | 0.18 | >0.999999 | 0.02 | 0.33 | 0.02 | 0.34 | >0.999999 | 0.02 | 0.29 | 0.02 | 0.29 | >0.999999 | | 0.02 | 0.27 | 0.02 | 0.27 | >0.999999 | | 0.63 | 14.58 | 0.61 | 14.25 | SS | |
| >0.999999 | 0.02 | 0.18 | 0.02 | 0.18 | >0.999999 | 0.03 | 0.38 | 0.02 | 0.38 | >0.999999 | 0.02 | 0.32 | 0.02 | 0.32 | >0.999999 | | 0.02 | 0.29 | 0.02 | 0.29 | 0.3835 | | 0.31 | 5.41 | 0.12 | 5.11 | PIR | |
| 0.6616 | 0.01 | 0.19 | 0.03 | 0.20 | >0.999999 | 0.01 | 0.34 | 0.03 | 0.34 | >0.999999 | 0.01 | 0.29 | 0.02 | 0.29 | >0.999999 | | 0.01 | 0.26 | 0.02 | 0.26 | >0.999999 | | 0.34 | 3.10 | 0.24 | 3.05 | TT | |
| 0.7924 | 0.04 | 0.17 | 0.07 | 0.19 | >0.999999 | 0.06 | 0.38 | 0.14 | 0.43 | >0.999999 | 0.06 | 0.33 | 0.13 | 0.37 | >0.999999 | | 0.06 | 0.30 | 0.13 | 0.33 | >0.999999 | | 0.01 | 0.07 | 0.01 | 0.07 | MOB_gl | |
| 0.7047 | 0.03 | 0.24 | 0.04 | 0.26 | >0.999999 | 0.03 | 0.37 | 0.03 | 0.35 | >0.999999 | 0.02 | 0.29 | 0.03 | 0.28 | >0.999999 | | 0.02 | 0.26 | 0.03 | 0.24 | >0.999999 | | 0.02 | 0.10 | 0.01 | 0.10 | MOB_gr | |
| >0.999999 | 0.03 | 0.21 | 0.04 | 0.21 | >0.999999 | 0.02 | 0.35 | 0.02 | 0.34 | >0.999999 | 0.02 | 0.29 | 0.02 | 0.28 | >0.999999 | | 0.02 | 0.26 | 0.02 | 0.25 | >0.999999 | | 0.28 | 3.46 | 0.32 | 3.35 | RHP | |
| 0.8837 | 0.04 | 0.18 | 0.04 | 0.19 | >0.999999 | 0.03 | 0.34 | 0.02 | 0.35 | >0.999999 | 0.02 | 0.29 | 0.01 | 0.29 | >0.999999 | | 0.02 | 0.26 | 0.01 | 0.26 | >0.999999 | | 0.19 | 5.21 | 0.27 | 5.04 | EC | |
| 0.6616 | 0.04 | 0.22 | 0.04 | 0.24 | >0.999999 | 0.03 | 0.37 | 0.02 | 0.37 | >0.999999 | 0.03 | 0.30 | 0.02 | 0.30 | >0.999999 | | 0.02 | 0.27 | 0.02 | 0.26 | >0.999999 | | 0.58 | 4.81 | 0.44 | 4.77 | CA1 | |
| 0.8628 | 0.04 | 0.22 | 0.04 | 0.24 | >0.999999 | 0.14 | 0.43 | 0.07 | 0.42 | >0.999999 | 0.11 | 0.35 | 0.05 | 0.34 | >0.999999 | | 0.10 | 0.31 | 0.05 | 0.30 | >0.999999 | | 0.40 | 3.03 | 0.39 | 3.04 | CA3 | |
| 0.6616 | 0.03 | 0.21 | 0.03 | 0.23 | >0.999999 | 0.02 | 0.40 | 0.02 | 0.40 | >0.999999 | 0.01 | 0.33 | 0.01 | 0.32 | >0.999999 | | 0.01 | 0.29 | 0.01 | 0.28 | >0.999999 | | 0.32 | 3.34 | 0.27 | 3.31 | DG | |
| 0.6616 | 0.02 | 0.23 | 0.03 | 0.25 | >0.999999 | 0.11 | 0.40 | 0.06 | 0.40 | >0.999999 | 0.09 | 0.33 | 0.05 | 0.32 | >0.999999 | | 0.08 | 0.29 | 0.05 | 0.28 | >0.999999 | | 0.06 | 0.31 | 0.07 | 0.31 | CA2 | |
| 0.7924 | 0.03 | 0.20 | 0.06 | 0.22 | >0.999999 | 0.03 | 0.36 | 0.03 | 0.35 | >0.999999 | 0.02 | 0.30 | 0.04 | 0.29 | >0.999999 | | 0.02 | 0.28 | 0.04 | 0.26 | >0.999999 | | 0.02 | 0.12 | 0.02 | 0.12 | MOB_mi | |
| 0.6616 | 0.02 | 0.21 | 0.01 | 0.20 | >0.999999 | 0.03 | 0.34 | 0.03 | 0.34 | >0.999999 | 0.03 | 0.28 | 0.03 | 0.29 | >0.999999 | | 0.03 | 0.25 | 0.03 | 0.26 | >0.999999 | | 0.41 | 6.84 | 0.37 | 6.84 | STR | |
| 0.9515 | 0.01 | 0.28 | 0.01 | 0.28 | >0.999999 | 0.01 | 0.33 | 0.01 | 0.34 | >0.999999 | 0.01 | 0.26 | 0.01 | 0.26 | >0.999999 | | 0.01 | 0.22 | 0.01 | 0.22 | >0.999999 | | 0.39 | 6.39 | 0.21 | 6.51 | MB | |
| 0.9158 | 0.02 | 0.25 | 0.04 | 0.26 | >0.999999 | 0.03 | 0.38 | 0.03 | 0.38 | >0.999999 | 0.02 | 0.30 | 0.02 | 0.30 | >0.999999 | | 0.02 | 0.26 | 0.01 | 0.26 | >0.999999 | | 1.69 | 15.15 | 2.02 | 14.40 | MY | |
