# Supplementary figures and images for: Apolipoprotein-E deficiency leads to brain network alteration characterized by diffusion MRI and graph theory
Source: Front Neurosci. 2023 Nov 21;17:1183312. doi: 10.3389/fnins.2023.1183312 (PMC10702609; doi:10.3389/fnins.2023.1183312)

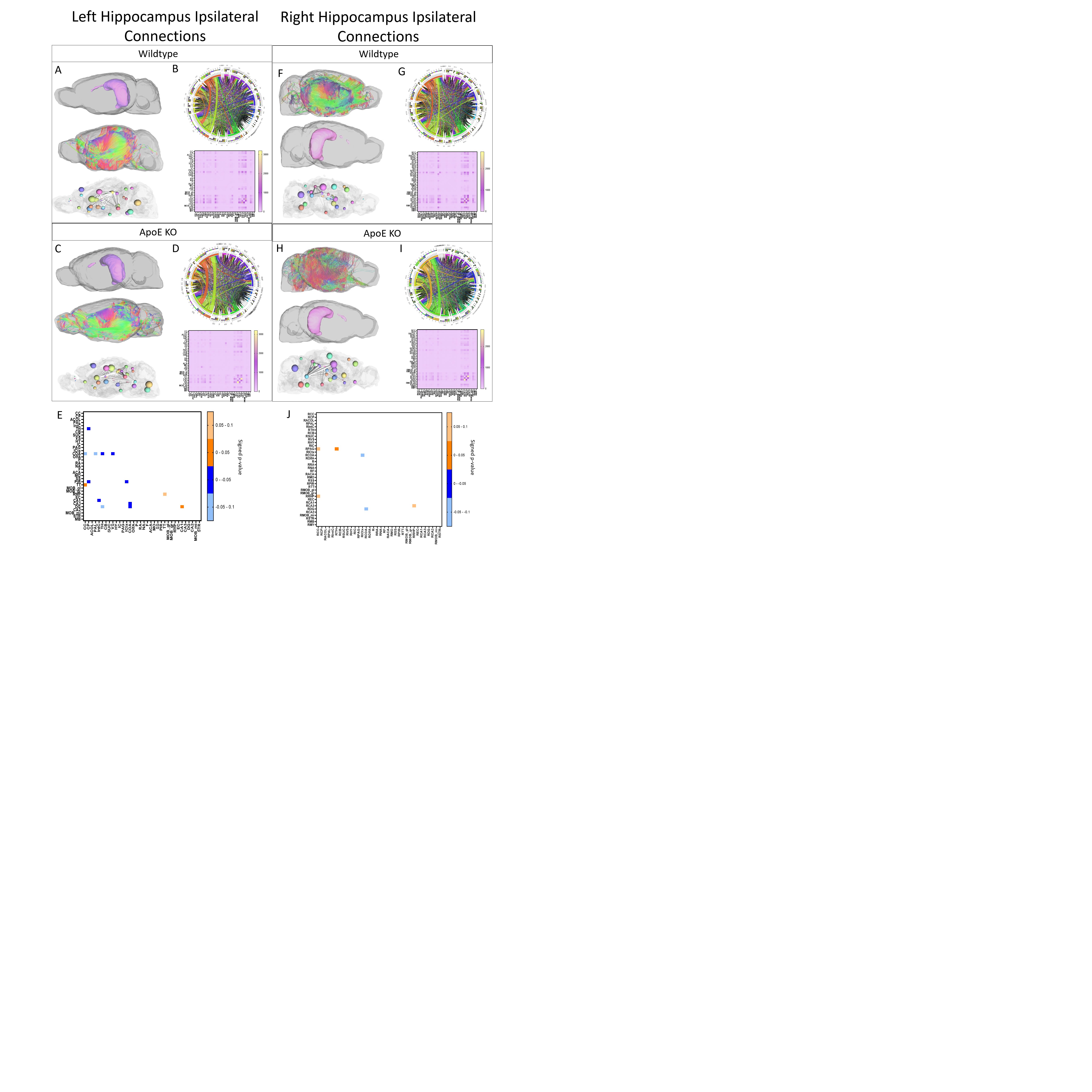

Supplement: Supplementary file 3 [file Image_1.JPEG]

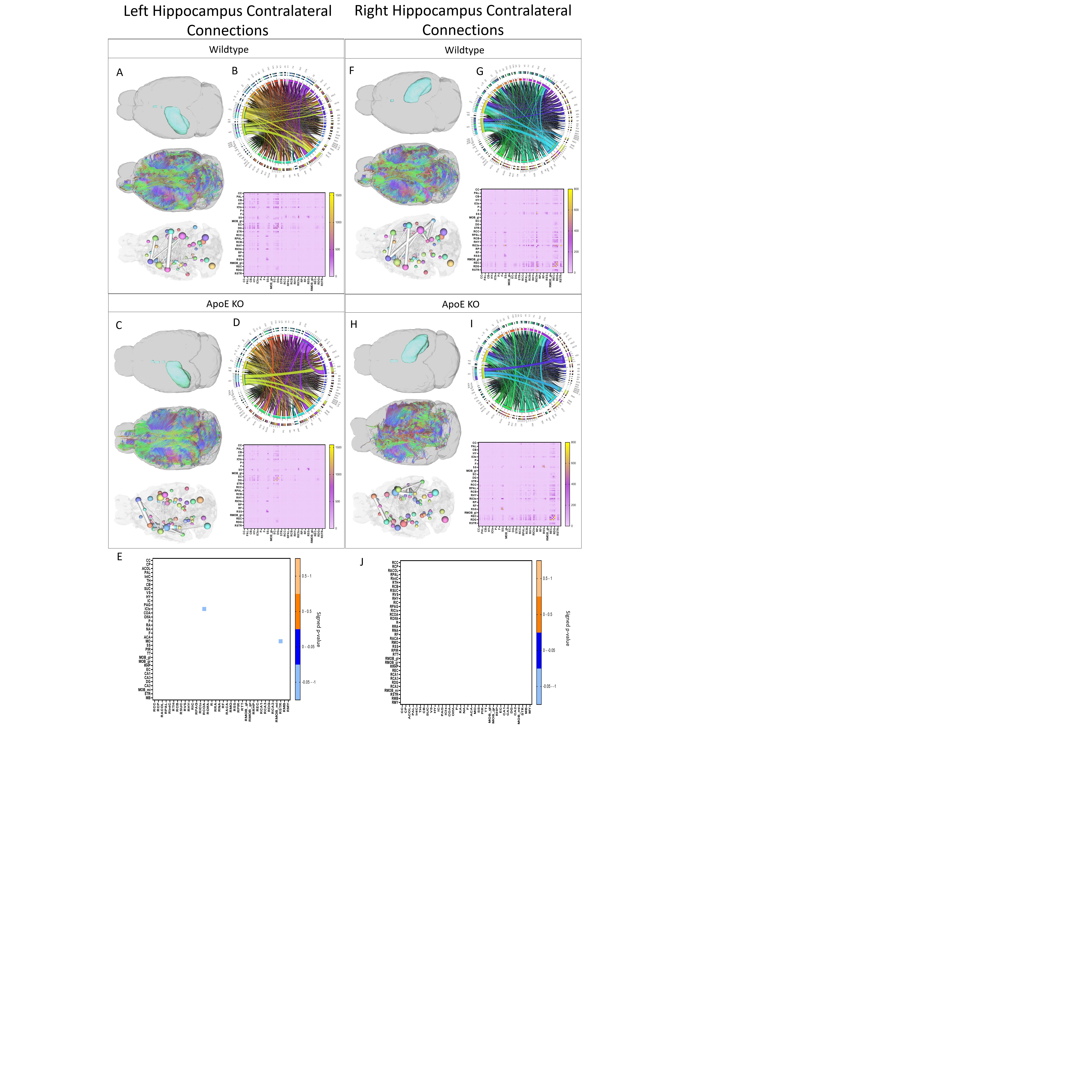

Supplement: Supplementary file 4 [file Image_2.JPEG]

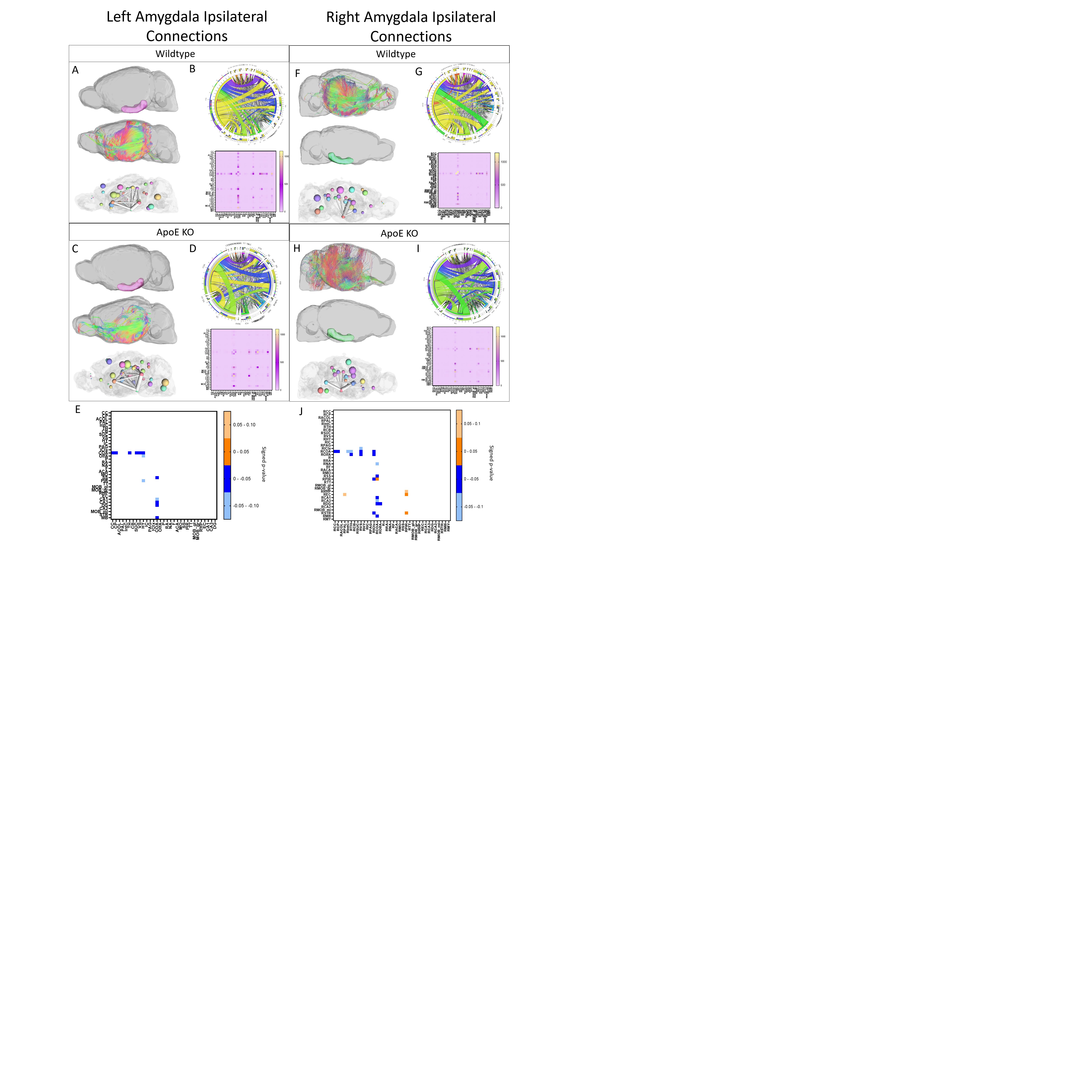

Supplement: Supplementary file 5 [file Image_3.JPEG]

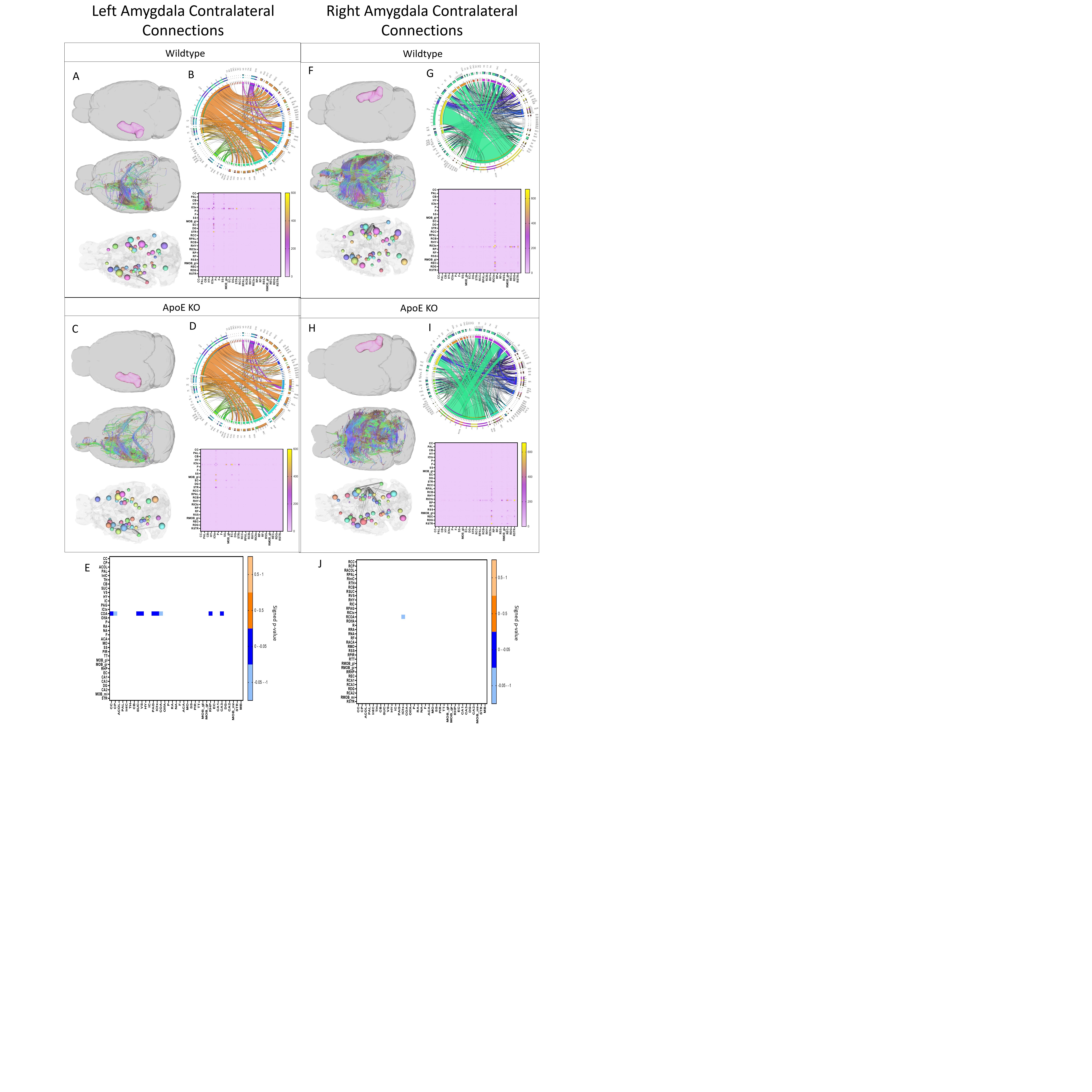

Supplement: Supplementary file 6 [file Image_4.JPEG]

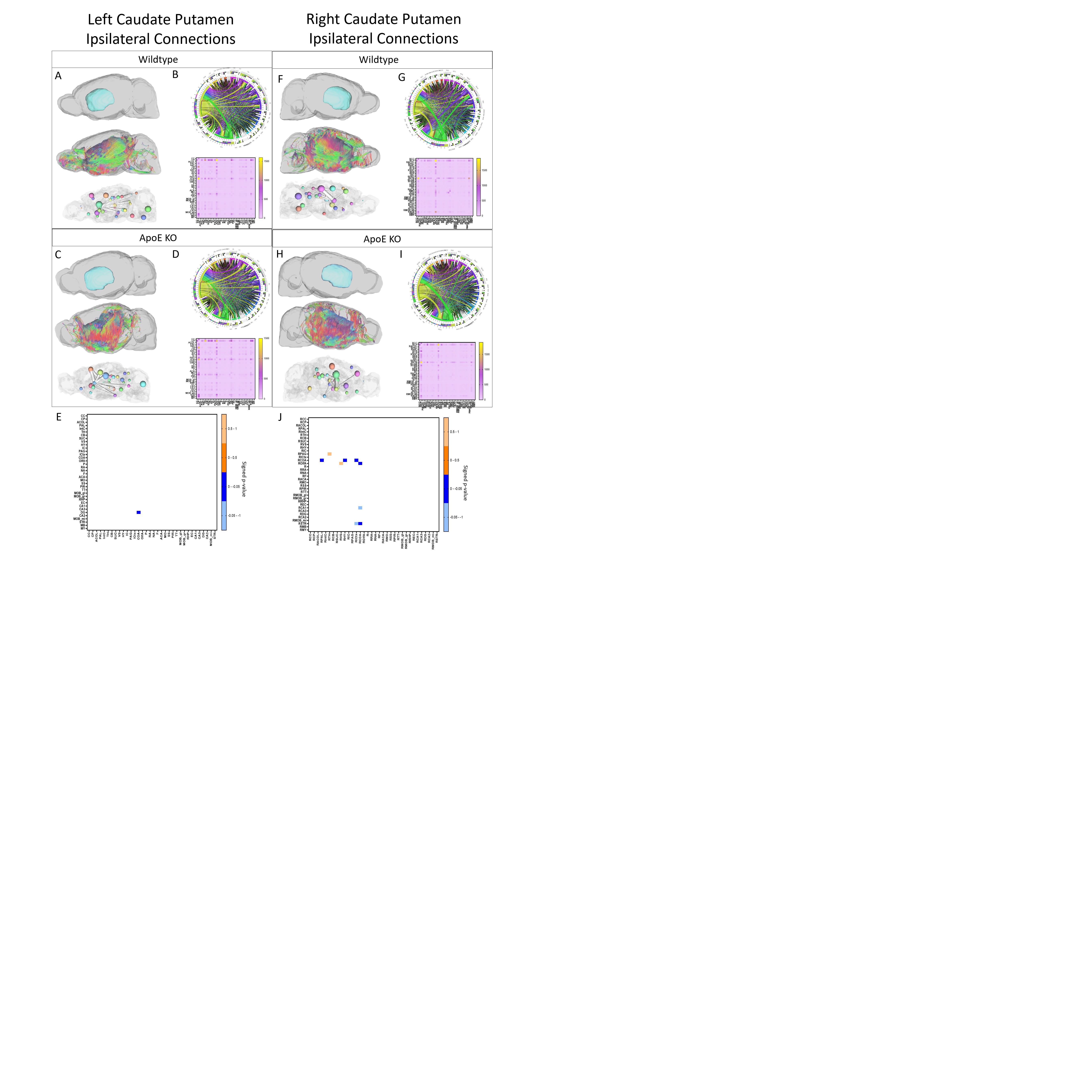

Supplement: Supplementary file 7 [file Image_5.JPEG]

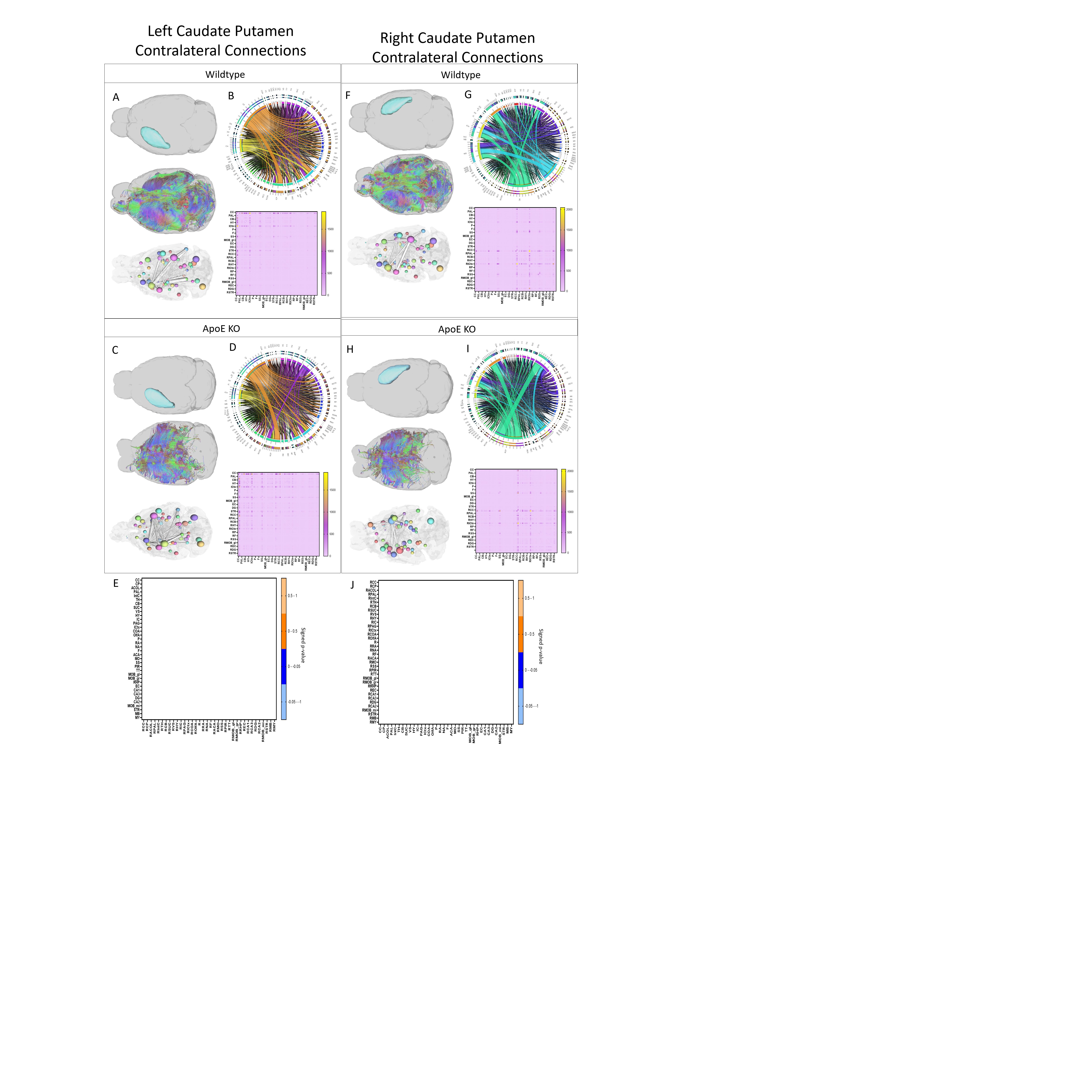

Supplement: Supplementary file 8 [file Image_6.JPEG]
